# The *higBA-*Type Toxin-Antitoxin System in IncC Plasmids Is a Mobilizable Ciprofloxacin-Inducible System

**DOI:** 10.1128/mSphere.00424-21

**Published:** 2021-06-02

**Authors:** Qin Qi, Muhammad Kamruzzaman, Jonathan R. Iredell

**Affiliations:** a Centre for Infectious Diseases and Microbiology, The Westmead Institute for Medical Research, The University of Sydney, Westmead, New South Wales, Australia; b Westmead Hospital, Westmead, New South Wales, Australia; University of Iowa

**Keywords:** *Enterobacteriaceae*, antibiotic resistance, plasmids, toxin-antitoxin systems

## Abstract

A putative type II toxin-antitoxin (TA) module almost exclusively associated with conjugative IncC plasmids is homologous to the *higBA* family of TA systems found in chromosomes and plasmids of several species of bacteria. Despite the clinical significance and strong association with high-profile antimicrobial resistance (AMR) genes, the TA system of IncC plasmids remains largely uncharacterized. In this study, we present evidence that IncC plasmids encode a bona fide HigB-like toxin that strongly inhibits bacterial growth and results in cell elongation in Escherichia coli. IncC HigB toxin acts as a ribosome-dependent endoribonuclease that significantly reduces the transcript abundance of a subset of adenine-rich mRNA transcripts. A glycine residue at amino acid position 64 is highly conserved in HigB toxins from different bacterial species, and its replacement with valine (G64V) abolishes the toxicity and the mRNA cleavage activity of the IncC HigB toxin. The IncC plasmid *higBA* TA system functions as an effective addiction module that maintains plasmid stability in an antibiotic-free environment. This *higBA* addiction module is the only TA system that we identified in the IncC backbone and appears essential for the stable maintenance of IncC plasmids. We also observed that exposure to subinhibitory concentrations of ciprofloxacin, a DNA-damaging fluoroquinolone antibiotic, results in elevated *higBA* expression, which raises interesting questions about its regulatory mechanisms. A better understanding of this *higBA-*type TA module potentially allows for its subversion as part of an AMR eradication strategy.

**IMPORTANCE** Toxin-antitoxin (TA) systems play vital roles in maintaining plasmids in bacteria. Plasmids with incompatibility group C are large plasmids that disseminate via conjugation and carry high-profile antibiotic resistance genes. We present experimental evidence that IncC plasmids carry a TA system that functions as an effective addiction module and maintains plasmid stability in an antibiotic-free environment. The toxin of IncC plasmids acts as an endoribonuclease that targets a subset of mRNA transcripts. Overexpressing the IncC toxin gene strongly inhibits bacterial growth and results in cell elongation in Escherichia coli hosts. We also identify a conserved amino acid residue in the toxin protein that is essential for its toxicity and show that the expression of this TA system is activated by a DNA-damaging antibiotic, ciprofloxacin. This mobile TA system may contribute to managing bacterial stress associated with DNA-damaging antibiotics.

## INTRODUCTION

Incompatibility group C (IncC) plasmids are large, conjugative, and broad-host-range plasmids found in diverse species of *Gammaproteobacteria* (1–3). They were first described in the early 1960s (3–6) and have been linked to the dissemination of high-profile antibiotic resistance genes that confer resistance to extended-spectrum β-lactams (ESBLs), carbapenems, and other clinically important antibiotics (7–9). Previously referred to as IncA/C plasmids (1, 10, 11), the IncC (formerly IncA/C_2_) and IncA (formerly IncA/C_1_) plasmids are now classified as separate plasmid incompatibility groups due to their ability to coexist stably in bacterial hosts (3, 12). A putative toxin-antitoxin (TA) system of IncC plasmids has been described (10, 13), but its function, regulation, and phenotypic effects on bacterial hosts remain largely uncharacterized. The putative toxin was suggested to be a host inhibition of growth (HigB)-like protein and a member of the RelE toxin superfamily (14). The putative antitoxin has been described as a HigA homolog and a xenobiotic response element (XRE)-like transcriptional regulator with a helix-turn-helix DNA-binding structure (15). When the toxin gene is present and expressed, disruption of the antitoxin gene in IncC plasmids results in a lethal phenotype for host cells (13). This TA system is also highly transcribed from IncC plasmids, which implies that the toxin contributes to the postsegregational killing of plasmid-free daughter cells (10, 15, 16).

Bacterial TA operons typically consist of two genes encoding a stable toxin that induces cell death or arrests growth and a labile antitoxin that neutralizes the toxin directly or indirectly. While the toxin is always a protein, the antitoxin can be protein or RNA based, and thus TA systems can be subtyped (types I to VII) based on the nature and mechanism of action of the antitoxin (17–19). The type II system, in which both the toxin and antitoxin are proteins, is the archetypical TA system and is probably the most common in bacteria (20). HigBA is a type II TA system of the RelBE superfamily found in conjugative plasmids (13, 21, 22) and in the chromosomes of well-studied and diverse bacterial species, including Mycobacterium tuberculosis (23), Pseudomonas aeruginosa (24), Escherichia coli (25), Caulobacter crescentus (26), Vibrio cholerae (27, 28), Proteus vulgaris (29), and Streptococcus pneumoniae (30). HigB toxin is a ribosome-dependent endoribonuclease (RNase) that cleaves translated mRNA substrates (31) and is sequestered by the cognate antitoxin protein HigA under normal growth conditions. The genomic organizations of *higBA* operons differ from those of most other TA operons in that most toxin genes (e.g., *relE*) are usually found downstream of their cognate antitoxin genes (e.g., *relB*), while *higB* toxin genes are always found upstream of *higA* antitoxin genes; *higBA* has been described as a *relBE* locus with an inverted gene order (25, 32). Homologs of HigB toxins exhibit endoribonuclease activities by cleaving specific mRNA codons in the A sites of ribosomes during translation. Whereas the closely related toxin RelB generally cleaves mRNA substrates upstream of purines in codons, HigB usually targets AAA codons (encoding lysine) in adenine-rich mRNA substrates, cutting between the second and third nucleotides, but it may also cleave AAA sequences that are out of frame *in vivo* (21).

In this study, we show that the *higBA*-type TA system found in IncC plasmids is an effective addiction module that displays key characteristics of chromosomal and plasmid-borne *higBA* homologs characterized in other bacterial species. The IncC toxin strongly inhibits bacterial growth by acting as a ribosome-dependent endoribonuclease that significantly reduces the transcript abundance of a subset of adenine-rich mRNA transcripts. The expression of this TA system is upregulated by treatment with the DNA-damaging antibiotic ciprofloxacin, which raises interesting questions about potential regulation mechanisms. This module is the only TA system that we found in the IncC plasmid backbone that is known to be associated with dangerous antimicrobial resistance (AMR) genes.

## RESULTS

### The *tad-ata* toxin-antitoxin system of IncC plasmids is a HigBA homolog.

The *tad* (toxin for addiction system) and *ata* (antitoxin for addiction system) of IncC plasmids (formerly known as IncA/C_2_) was previously predicted to be a *higBA*-type TA operon (10, 13). TAfinder (33) identifies this as a type II TA system in the complete sequence of IncC plasmid pEc158 (GenBank accession no. KY887596.1), with a RelE-type toxin and an XRE family protein antitoxin, which we confirmed with a BLASTP search. The unusual genetic organization of the TA system in the IncC plasmid backbone, with the toxin gene found upstream of the antitoxin gene, is opposite to those of canonical type II TA systems, including other members of the RelBE and ParDE superfamilies. Interestingly, HigBA TA systems in other bacterial species are known to exhibit the same gene order as that found in IncC plasmids (28, 34).

**FIG 1:**
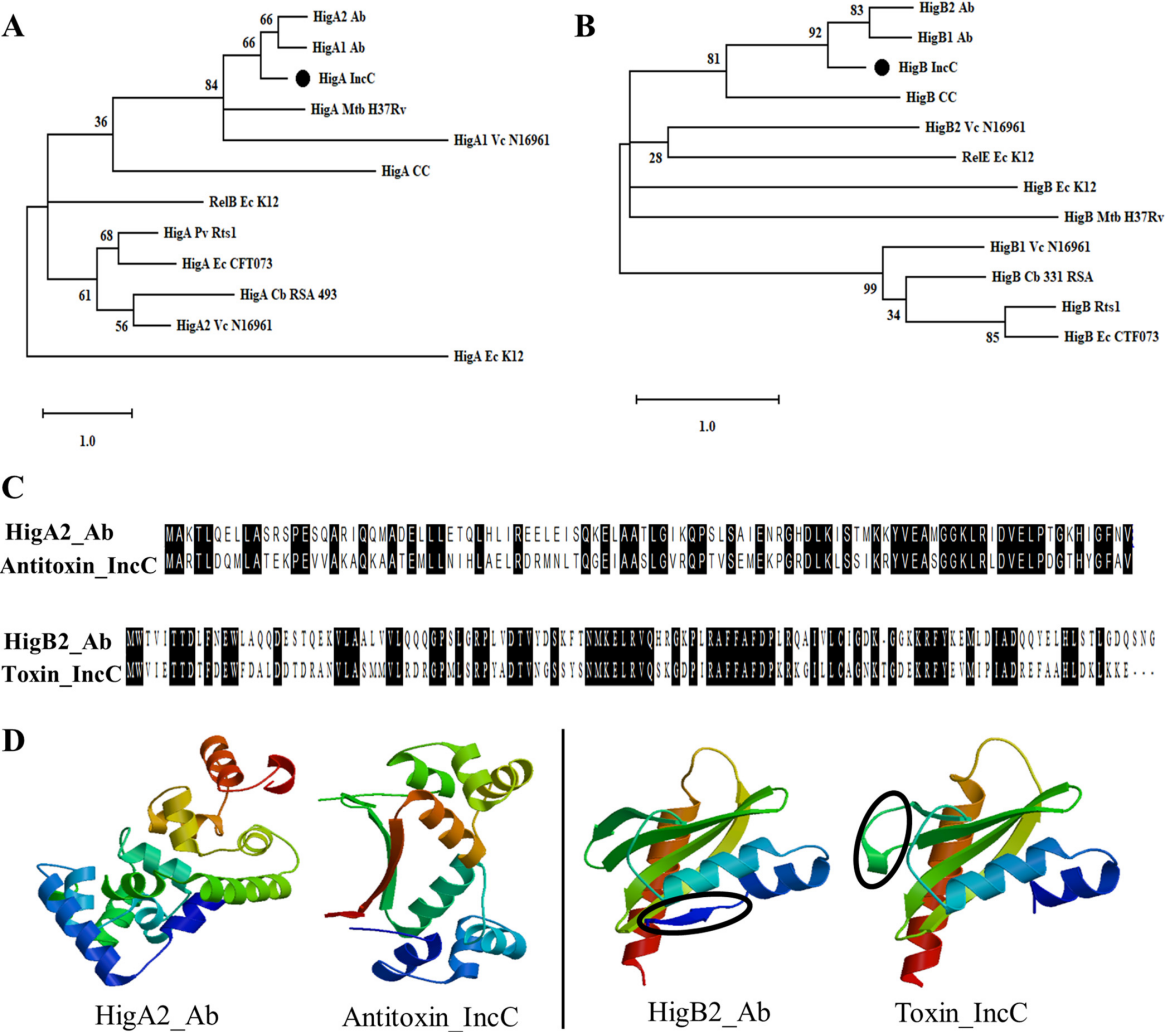
Phylogenetic analysis, amino acid sequence alignments, and prediction of tertiary structures for IncC HigB toxin and HigA antitoxin proteins. (A and B) Phylogenetic trees for HigA/RelB antitoxins (A) and HigB/RelE toxins (B) from 7 bacterial species were constructed by the maximum-likelihood method, with a bootstrap value of 500. The percentage of trees in which the associated taxa clustered together is shown next to the branches. The homologs for HigB and HigA found in IncC plasmids are indicated by the black circles. The TA systems from the different bacterial species are represented using the following abbreviations: Vc, Vibrio cholerae; Cb, Coxiella burnetii; Pv, Proteus vulgaris; Ec, E. coli; Ab, Acinetobacter baumannii; Mtb, Mycobacterium tuberculosis; and Cc, Caulobacter crescentus. (C) Amino acid sequence alignments of the putative antitoxin and toxin of the IncC plasmid with the HigA antitoxin and HigB toxin of A. baumannii plasmid pAB120. Identical amino acids are shaded in black. (D) Modeled tertiary structures for the putative antitoxin and toxin of the IncC plasmid and their homologs found in A. baumannii plasmid pAB120. The structural differences between the toxin proteins are marked with black circles.

Using phylogenetic analysis, we compared the amino acid sequences of the putative toxin and antitoxin found in IncC plasmids with those of other HigBA and RelBE TA systems that have been experimentally characterized. The putative TA proteins of IncC plasmid appear most similar to the HigBA TA system proteins of Acinetobacter baumannii plasmid pAB120 (Fig. 1A and B), with 51% amino acid sequence identity for both the antitoxin and toxin (Fig. 1C). The two sets of homologs have similar predicted secondary structures (Fig. S1), but some differences can be observed in the modeled tertiary structures (Fig. 1D). The antitoxin of IncC plasmid TA has two β-sheets, whereas the HigA antitoxin of A. baumannii has none. The HigB toxin of A. baumannii has one small β-sheet at the N-terminal end, whereas a twisted hinge was observed between the first and second β-sheets of the IncC HigB homolog.

10.1128/mSphere.00424-21.1 FIG S1 (A and B) Predicted protein secondary structures of HigA_IncC and HigA2_Ab (A) and HigB_IncC, HigB2_Ab, and HigB_IncC (G64V) (B). The predicted α-helix (H, purple box), β-strand (E, yellow box), and coil (C, gray line) are shown for the structure/prediction (Str./Pred.) at each amino acid (AA) position. The numbers indicate the positions of amino acids in the protein from the N to the C terminus. Download FIG S1, TIF file, 0.2 MB. Copyright © 2021 Qi et al. 2021 Qi et al. https://creativecommons.org/licenses/by/4.0/ This content is distributed under the terms of the Creative Commons Attribution 4.0 International license.

### Distribution of the putative HigBA-like TA system in GenBank.

The putative IncC plasmid *higBA* TA system comprises a 351-bp open reading frame for the putative toxin and a downstream 303-bp putative antitoxin. A BLASTN search for the nucleotide sequences of the complete TA system (the coding regions of both the toxin and antitoxin genes) identified 462 hits, with 100% length coverage and at least ∼89% nucleotide sequence identity (see Table S1 in the supplemental material). Further examination reveals this *higBA*-like TA system to be exclusive to plasmids of different *Enterobacteriaceae* species. Of all the plasmids that were identified by PlasmidFinder (35), 97.2% (449/462) were IncC plasmids (Fig. S2 and Table S1).

10.1128/mSphere.00424-21.2 FIG S2 Distribution of the *higBA* TA system in GenBank sequences. Download FIG S2, TIF file, 0.08 MB. Copyright © 2021 Qi et al. 2021 Qi et al. https://creativecommons.org/licenses/by/4.0/ This content is distributed under the terms of the Creative Commons Attribution 4.0 International license.

10.1128/mSphere.00424-21.4 TABLE S1 Distribution of the *higBA* TA system in GenBank. Download Table S1, XLSX file, 0.04 MB. Copyright © 2021 Qi et al. 2021 Qi et al. https://creativecommons.org/licenses/by/4.0/ This content is distributed under the terms of the Creative Commons Attribution 4.0 International license.

### The *higBA-*like operon of IncC plasmids is a toxin-antitoxin system.

We observed two main variants of the *higB-*like toxin gene, here referred to as variants 1 and 2, that differ by 9 synonymous mutations (Table S2). Their wide distribution in the GenBank sequence data for IncC plasmids suggests strong selection for conserved amino acid residues in the HigB-like toxin protein. IncC plasmids are classified as type 1, 2, or 1/2-hybrid based on the structures of their backbones, and their differences are well described in the literature (3, 36–38). We collated a list of 63 sequenced plasmids from several studies with known IncC plasmid types (7, 36–39) and examined whether there is a correlation between toxin gene variants and IncC plasmid types (Table S3). Our analysis revealed variant 1 to be the more prevalent variant; it can be found in all three types of IncC plasmids. In contrast, variant 2 was observed only in type 1 IncC plasmids, not in the other two types.

10.1128/mSphere.00424-21.5 TABLE S2 Nucleotide differences in the two variants of the IncC *higB* toxin gene. Download Table S2, DOCX file, 0.01 MB. Copyright © 2021 Qi et al. 2021 Qi et al. https://creativecommons.org/licenses/by/4.0/ This content is distributed under the terms of the Creative Commons Attribution 4.0 International license.

10.1128/mSphere.00424-21.6 TABLE S3 Distribution of two *higB-*like toxin gene variants in a selection of plasmids with known IncC backbone types. Download Table S3, XLSX file, 0.02 MB. Copyright © 2021 Qi et al. 2021 Qi et al. https://creativecommons.org/licenses/by/4.0/ This content is distributed under the terms of the Creative Commons Attribution 4.0 International license.

**FIG 2:**
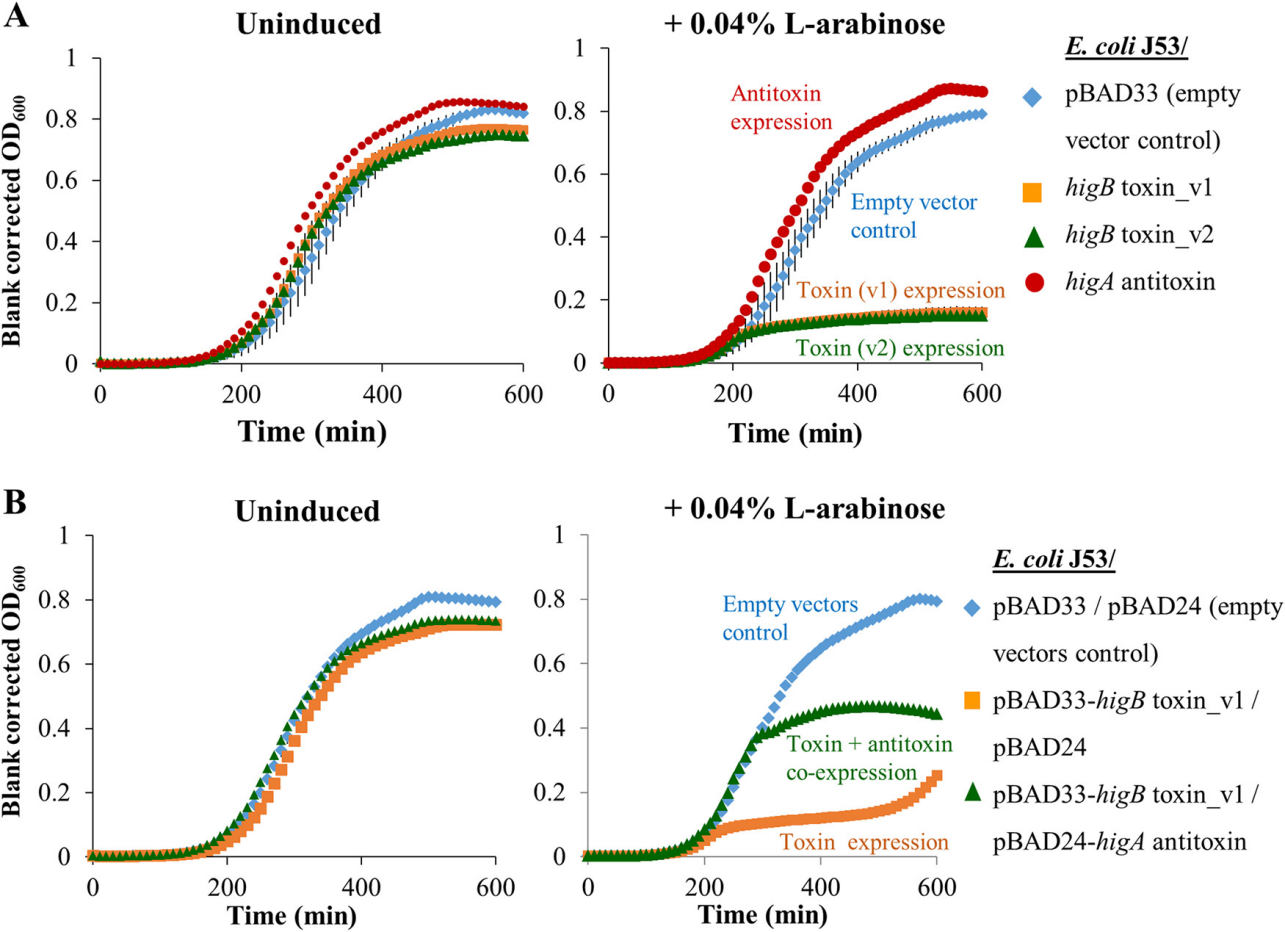
Overexpression of the IncC *higB-*like toxin gene strongly inhibits growth, while coinduction of the *higA-*like antitoxin gene restores the growth rate of the E. coli J53 host. (A) The slow growth of the E. coli J53 host strain in which variants 1 (right panel, orange line) and 2 (right panel, green line) of the *higB-*like toxin gene were overexpressed in *trans* compared to their expression in the uninduced reference groups for the same strains (left panel) demonstrates the strong deleterious effects of the putative toxin on host growth. (B) The E. coli J53 host strain in which both the *higB-*like toxin gene and the *higA-*like antitoxin gene were overexpressed in *trans* (green line) grew faster than the strain in which only the *higB-*like gene was overexpressed (orange line), suggesting that the *higA-*like gene codes for the cognate antitoxin gene of the HigB-like toxin.

To demonstrate the bona fides of the *higB-*like gene as a toxin, the coding regions of *higB* variant 1 (v1) and v2 were cloned downstream of an l-arabinose-inducible promoter (P*_ara_*) in the low-copy-number plasmid pBAD33-Gm (gentamicin-resistant expression vector with the p15A replicon; subsequently referred to as pBAD33). The growth curves for E. coli J53/pBAD33-*higB* were obtained for growth in LB broth with and without 0.04% l-arabinose induction at 37°C. In Fig. 2A, the growth curves show that induction of in *trans* expression of either variant of the *higB-*like toxin gene strongly inhibited the growth of the E. coli J53 host compared to that of the uninduced controls. To demonstrate the growth rescue effect of the putative antitoxin in this TA system, the *higA-*like gene was cloned downstream of P*_ara_* in the pBAD24 expression vector (ampicillin-resistant plasmid with the pBR322 replicon). We then generated an E. coli J53 strain that carries both the pBAD33-*higB* and pBAD24-*higA* plasmids, which are compatible in J53 grown in LB medium that contains gentamicin and ampicillin. During the exponential and early stationary phases, we observed that the simultaneous expression of *higA* and *higB* in J53/pBAD33-*higB*/pBAD24-*higA* resulted in a higher growth rate than that of the J53/pBAD33-*higB*/pBAD24 strain, in which only *higB* was expressed (Fig. 2B), indicating rescue from the effects of HigB by *higA* expression in *trans*. The lower final optical density (OD) reached by the l-arabinose-induced J53/pBAD33-*higB*/pBAD24-*higA* strain in stationary phase compared to that reached by the induced J53/pBAD33/pBAD24 control strain might potentially be due to the metabolic burden of *higB* and *higA* overexpression, as well as potential imbalances in the abundances of HigB and HigA, which were expressed in *trans*.

### The *higBA-*like operon of IncC plasmids is an effective addiction system.

TA systems typically promote the retention of TA-bearing plasmids in the absence of antibiotic selection pressure. To establish that the *higBA-*like operon in IncC plasmids is an addiction module, we cloned the TA operon with its promoter region and ribosome binding site (RBS) into a low-copy-number vector, pACYC184 (chloramphenicol resistant), to generate pACYC184-*higBA*. pACYC184 with and without *higBA* was used to transform an E. coli J53 host strain that was chromosomally tagged with a green fluorescence protein gene (*gfpuv*). The J53-*gfpuv*/pACYC184 control and J53-*gfpuv*/pACYC184-*higBA* (with *higB* v1 or v2) strains were passaged for 70 generations in antibiotic-free LB medium. All three strains showed similar growth kinetics in antibiotic-free LB medium (data not shown), which suggests that *higBA* expression from pACYC184 did not introduce unintended growth defects.

**FIG 3:**
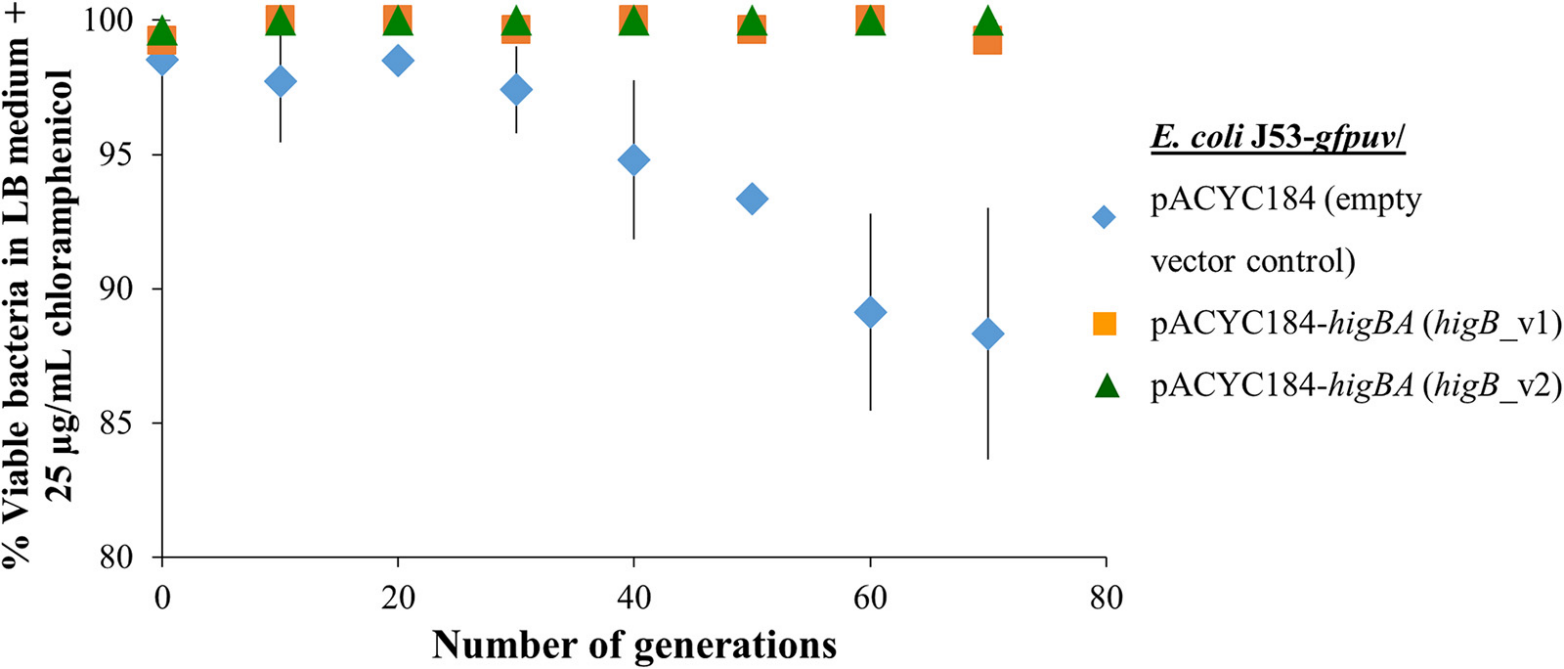
The IncC plasmid TAS functions as an effective addiction module that promotes plasmid stability in an antibiotic-free growth environment. E. coli J53-*gfpuv* strains that carried the low-copy-number pACYC184 vectors with and without the *higBA-*like operons from IncC plasmid were propagated in antibiotic-free LB broth for 70 generations. The J53-*gfpuv*/pACYC184 control lineage (blue line) gradually lost plasmids during the passaging. In contrast, nearly full retention of the pACYC184-*higBA*_v1 (orange line) and pACYC184-*higBA*_v2 (green line) plasmids was observed under the same experimental conditions in J53-*gfpuv* lineages that carried those plasmids.

During the plasmid stability assay, serial transfers were performed by diluting saturated cultures of each strain 1:1,000 in fresh antibiotic-free LB medium. Ten generations were estimated to have elapsed between each serial transfer. At each sampling point, 93 single J53-*gfpuv* cells were randomly captured and sorted into antibiotic-free LB medium by fluorescence-activated cell sorting (FACS). Plasmid retention was assessed by challenging the sorted cells with chloramphenicol after overnight incubation at 37°C. By the end of the plasmid stability assay, 11.7% ± 4.7% (mean ± standard error of the mean [SEM], *n* = 3) of the sampled cells in the endpoint populations of the J53-*gfpuv*/pACYC184 lineage lost their plasmids (Fig. 3). In contrast, the J53-*gfpuv*/pACYC184-*higBA_*v1 and pACYC184-*higBA_*v2 populations maintained their plasmids, with retention rates of 99.3% ± 0.4% and 100%, respectively. This is consistent with our expectation that the *higBA-*like operon of IncC plasmids contributes to plasmid maintenance in an antibiotic-free environment and functions as an addiction module.

### Inactivating mutation G64V abolishes the phenotypic effects of *higB* induction.

**FIG 4:**
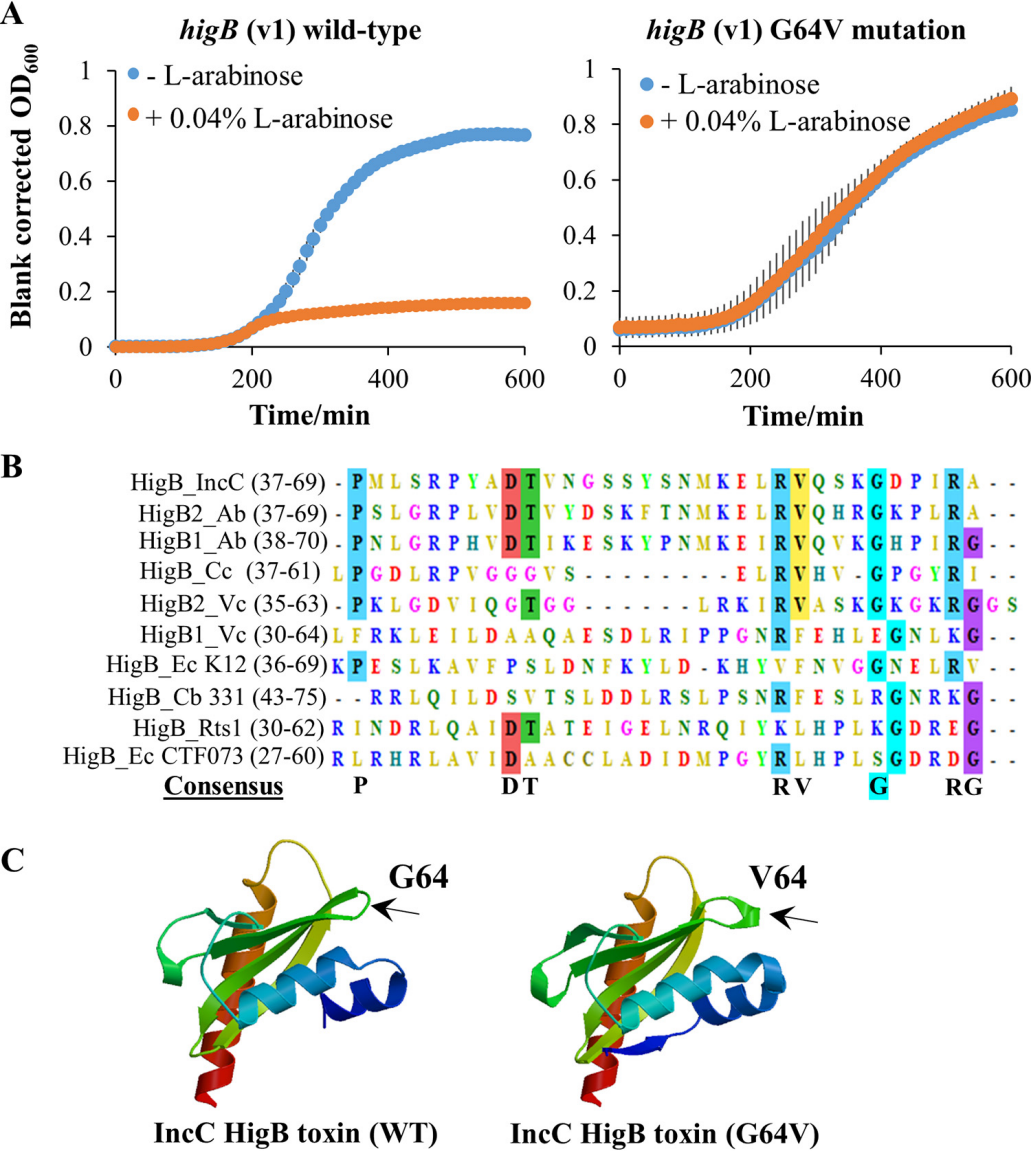
Amino acid residue G64 in the coding region of the HigB-like toxin is conserved and essential for its toxicity. (A) The inactivating mutation G64V in the coding region of the *higB-*like toxin gene fully offsets the deleterious effects of overexpressing the toxin gene on J53 host strain growth. (B) Alignment of the amino acid sequences of the HigB toxins. It was found that G64 (highlighted in cyan in the alignment and in the consensus sequence) of IncC HigB is well conserved in the other HigB toxins found in the plasmids and chromosomes of different species. (C) The change in modeled protein tertiary structures of the HigB toxin of the IncC plasmid after the G64V mutation is indicated by the black arrows.

Nine escape mutant strains of J53/pBAD33-*higB*_v1 that grew significantly faster than their ancestral strain after 16 h of *higB* expression in *trans* were isolated by streaking saturated bacterial cultures that contained putative escape mutants on gentamicin selective medium. Escape mutants were detected in 9 out of 27 independent l-arabinose-induced cultures after 16 h of growth on four different days, implying a strong selection for inactivation of the *higB*-like toxin gene. Sanger sequencing of the plasmids extracted from seven of these evolved J53/pBAD33-*higB*_v1 strains revealed nonsynonymous or indel (in-frame or frameshift) mutations in the coding region of *higB* in six strains and disruption in the l-arabinose-inducible promoter region in one strain (Table S4). In this study, we focused on the G64V mutation, because it was the only mutation we isolated that resulted in the substitution of a single amino acid. More importantly, the G64V mutation abolished the growth defect when *higB* (G64V) was overexpressed in *trans* (Fig. 4A). In contrast to the strains that carried wild-type *higB*, J53/pBAD33-*higB* (G64V) showed negligible differences in growth rates regardless of whether the l-arabinose inducer was present.

10.1128/mSphere.00424-21.7 TABLE S4 Mutations affecting the promoter or coding region of the IncC *higB* toxin gene in pBAD33-*higB_*v1 plasmids extracted from escape mutant isolates of E. coli J53/pBAD33-*higB_*v1. Download Table S4, DOCX file, 0.01 MB. Copyright © 2021 Qi et al. 2021 Qi et al. https://creativecommons.org/licenses/by/4.0/ This content is distributed under the terms of the Creative Commons Attribution 4.0 International license.

By analyzing the protein sequences of various HigB toxins, we found that the glycine residue at the 64th position in the IncC HigB toxin is very well conserved in HigB homologs found in the chromosomes and plasmids of different bacterial species (Fig. 4B). The conserved glycine residue was present at the 64th position in IncC HigB, A. baumannii pAB120 plasmid HigB2, and E. coli K-12 chromosomal HigB; in the 65th position of A. baumannii HigB1, E. coli CTF073 HigB, C. crescentus chromosomal HigB, and V. cholerae HigB2; the 58th position of Rts1 plasmid HigB from Proteus vulgaris; the 59th position of V. cholerae HigB1; and the 71st position of C. burnetii HigB protein. IncC HigB with and without the G64 mutation did not show any difference in the predicted secondary structures (Fig. S1B), but small changes appeared in the modeled tertiary structures (Fig. 4C). The valine residue subtly changes folding at this position, and this predicted alteration in tertiary structure rendered the mutant variant nontoxic, possibly because G64V damaged the catalytic activity of a key amino acid at the active site of the toxin protein.

### IncC HigB toxin reduces the abundance of a subset of AAA-rich mRNA transcripts, and glycine residue G64 in HigB is essential for mRNA cleavage.

HigB toxins in several bacterial species are known to cleave mRNA substrates that are rich in AAA codons, such as *lpp* and *ompA* transcripts (21, 25, 28, 40). The *lpp* gene codes for the most abundant lipoprotein in E. coli (41), while *ompA* codes for a major protein in the outer membrane (42). Both genes are nonessential housekeeping genes that are stable and abundantly expressed under normal growth conditions (43, 44) and have been frequently used as mRNA substrates to demonstrate the endoribonuclease activities of HigB and RelE homologs.

Using 0.1% l-arabinose, we induced the ectopic expression of the IncC *higB* (v1 wild-type) gene and the mutant variant containing the inactivating mutation G64V from the pBAD33-Gm expression vector in E. coli J53. After 3 h of ectopically expressing *higB*, we extracted the total RNA from the induced and uninduced strains and quantified the transcript abundances of *lpp* and *ompA* in the induced strain by real-time quantitative PCR (qRT-PCR) relative to abundances in the J53/pBAD33 vector control, applying the geNorm algorithm (45) to evaluate the expression stability of five candidate internal reference genes. These included two nontranslated genes (16S rRNA and the tRNA-like domain of the transfer mRNA [tmRNA] *ssrA*) and three essential genes (*rpoB*, *gyrB*, and *rho*). Their gene products and cellular roles are described in the supplemental material (Table S5). We found that 16S rRNA, *rpoB*, and *gyrB* formed the most stable trio, and their average expression levels were used to normalize the expression levels of the target genes (Fig. S3).

10.1128/mSphere.00424-21.3 FIG S3 Expression stability of five candidate internal reference genes for qRT-PCR assays involving unopposed expression of the IncC toxin gene. For qRT-PCR assays that involve overexpression of the IncC *higB* toxin gene, the GeNorm algorithm (13) was applied to assess the average expression stability of five candidate internal reference genes. 16S rRNA, *rpoB*, and *gyrB* were found to be the most stable combination, whose average expression was used for the normalization of gene expression levels of target genes (*lpp*, *ompA*, the mRNA-like and tRNA-like domains of *ssrA*). Download FIG S3, TIF file, 0.08 MB. Copyright © 2021 Qi et al. 2021 Qi et al. https://creativecommons.org/licenses/by/4.0/ This content is distributed under the terms of the Creative Commons Attribution 4.0 International license.

10.1128/mSphere.00424-21.8 TABLE S5 Oligonucleotide primers used in this study. Download Table S5, DOCX file, 0.02 MB. Copyright © 2021 Qi et al. 2021 Qi et al. https://creativecommons.org/licenses/by/4.0/ This content is distributed under the terms of the Creative Commons Attribution 4.0 International license.

**FIG 5:**
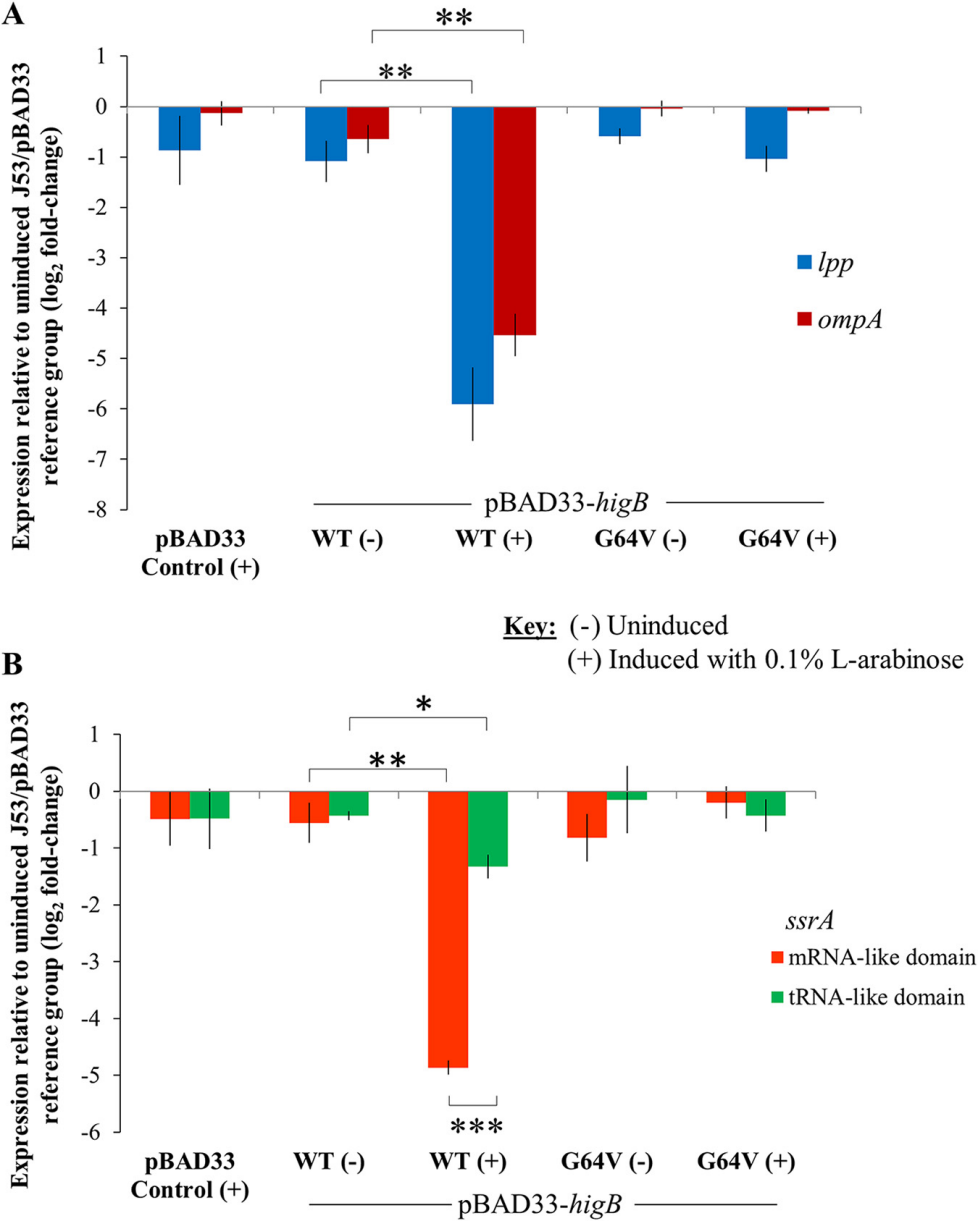
The HigB-like toxin of IncC plasmids depletes the transcript abundance of a subset of translated adenine-rich RNA substrates. (A) Overexpression of the *higB* toxin gene in the J53 host strain in *trans* strongly reduces the expression levels of both *lpp* and *ompA* relative to those in the uninduced groups for the same J53/pBAD33-*higB* strain (**, *P* < 0.01). The nonsynonymous mutation G64V in the *higB* toxin gene abolishes the downregulation of these genes. (B) Overexpression of the *higB* toxin gene in the J53 host strain in *trans* strongly reduces the relative transcript abundance of the mRNA-like domain of the transfer mRNA *ssrA* compared to that of the untranslated tRNA-like domain of the same gene (***, *P* < 0.001). Overexpressing *higB* that contains the G64V mutation has negligible effects on the relative transcript abundances of both domains of *ssrA*.

As shown in Fig. 5A, a significant reduction in *lpp*’s relative expression level (log_2_ fold change from that in the uninduced pBAD33 vector reference group) was observed in the *higB* wild-type expression group compared to that in the uninduced group for the same strain (Welch’s two-sample *t* test, *t *= 5.77, df = 3.14, *P* < 0.01 [**]). A similar reduction was seen for the relative expression level of *ompA* in the wild-type *higB* expression group compared to that in the uninduced group (Welch’s two-sample *t* test, *t *= 7.75, df = 3.48, *P* < 0.01 [**]).

To test whether the endoribonuclease activity of HigB is translation dependent, we quantified the transcript abundance of the tmRNA *ssrA*, which has a nontranslated tRNA-like domain, as well as a translated mRNA-like domain (46). During *trans*-translation, the *ssrA* tmRNA plays an important role in recycling stalled ribosomes in E. coli; the translated mRNA-like domain encodes a 10-amino-acid polypeptide (ANDENYALAA) that is attached to the C terminus of stalled peptides to facilitate their disassembly from stalled ribosomes. When the chromosomal *higB* toxin gene from M. tuberculosis was overexpressed in E. coli, sites within the translated mRNA-like domain of *ssrA* were cleaved, while the nontranslated tRNA-like domain was not affected, as shown by Northern blotting analysis (23).

We targeted two segments of *ssrA* by designing two sets of qRT-PCR primers that amplify different regions of the same gene. One amplicon spans the mRNA-like domain, while the other targets a segment of the tRNA-like domain. Figure 5B shows that the relative expression level of the amplicon spanning the mRNA-like domain decreased in the induced wild-type toxin group compared to that in the uninduced group of the same strain. The downregulation in relative expression was similar in magnitude to those observed for *lpp* and *ompA* when *higB* was overexpressed. Interestingly, the downregulation for the tRNA-like domain was significantly smaller than that of the mRNA-like domain (Welch’s two-sample *t* test, *t *= 14.5, df = 3.25, *P* < 0.001 [***]).

Notably, the G64V mutation abolished the effects of *higB* overexpression by depleting the relative expression levels of the three adenine-rich RNA substrates that we quantified (Fig. 5A and B), so the expression levels of *lpp*, *ompA*, and the mRNA-like domain of the *ssrA* tmRNA in the *higB* (G64V) overexpression group were similar to those in the uninduced *higB* and pBAD33 empty vector reference groups. This provides further evidence that the G64 residue in HigB is essential for the endoribonuclease activity of the toxin protein.

### Unopposed expression of the IncC *higB* toxin gene results in cell elongation.

**FIG 6:**
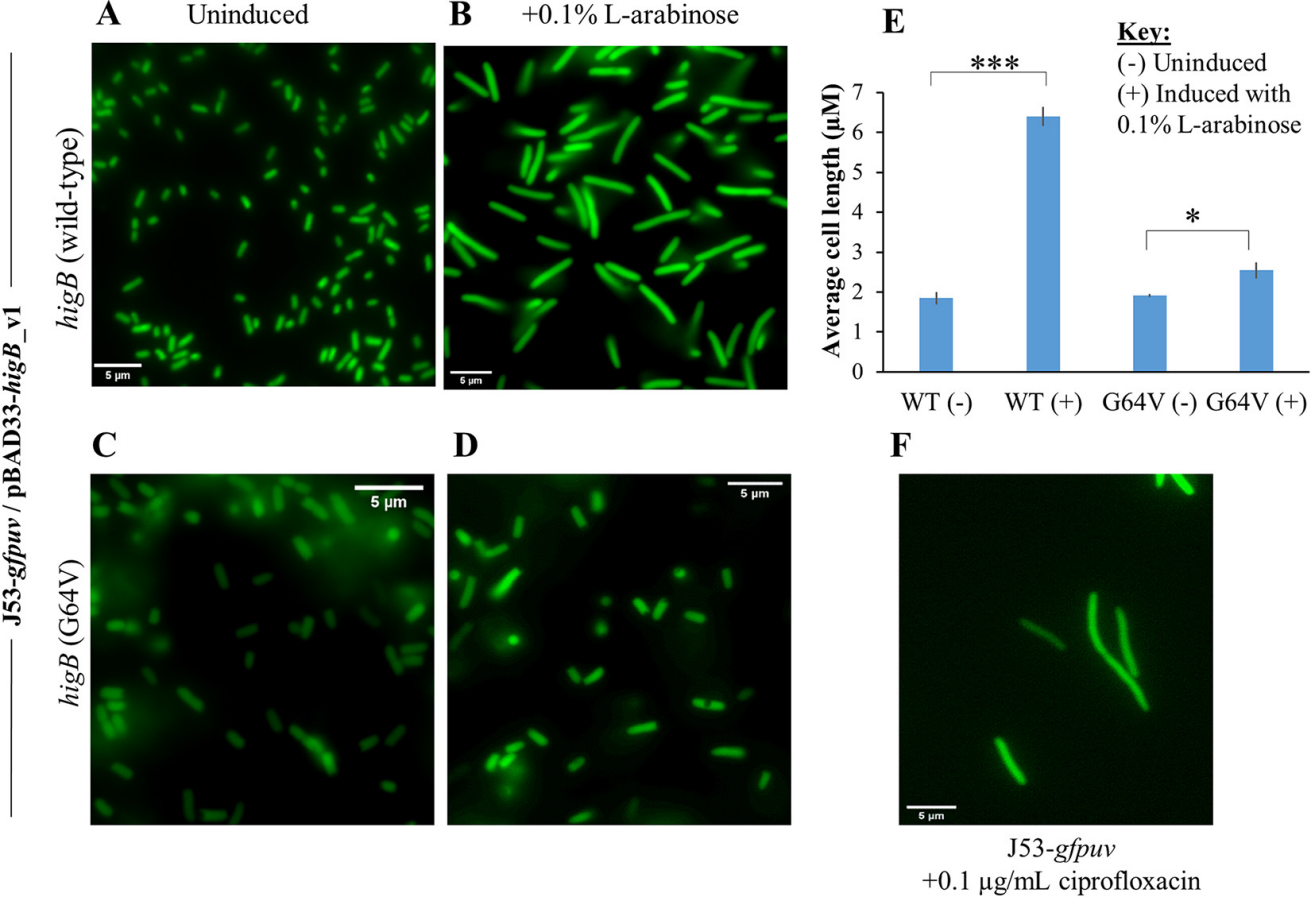
Overexpression of the IncC HigB toxin resulted in cell elongation of the E. coli J53-*gfpuv* host strain. (A and B) Overexpression of the wild-type *higB* toxin gene resulted in elongation of the J53-*gfpuv* host cells compared to those of the uninduced control. (C and D) Overexpressing the *higB* toxin gene that contains the G64V mutation significantly reduced the extent of cell elongation. (E) Unopposed expression of wild-type *higB* resulted in a significant increase in average cell length (***, *P* < 0.001). Although *higB* G64V overexpression also caused a statistically significant increase in cell length (*, *P* = 0.0317), the extent of cell elongation was greatly reduced. (F) HigB-induced cell elongation shows similarity to phenotypic changes observed in J53-*gfpuv* treated with ciprofloxacin.

To test the morphological effects of unopposed *higB* expression on E. coli host cells, we transformed the chromosomally fluorescence-tagged J53-*gfpuv* strain with the pBAD33-*higB*_v1 plasmid and induced *higB* expression with 0.1% l-arabinose. Compared to that in the uninduced J53-*gfpuv*/pBAD33-*higB*_v1 reference group, unopposed *higB* expression in J53-*gfpuv*/pBAD33-*higB*_v1 resulted in significant cell elongation when observed under a fluorescence microscope (Fig. 6A and B). Cell elongation was greatly reduced (Fig. 6C to E) when *higB* G64V was induced with 0.1% l-arabinose (Fig. 6D) relative to that in the uninduced strain (Fig. 6C), consistent with our expectation that the G64V inactivating mutation abolishes the phenotypic effects of the HigB toxin.

At a phenotypic level, the elongated cell morphology shows similarity to that of ciprofloxacin-treated J53-*gfpuv* (Fig. 6F), which is the result of DNA damage due to inhibition of DNA gyrase and topoisomerase IV (47). When the SOS response is activated by DNA damage, cleavage of LexA results in upregulation of the cell division inhibitor gene *sulA*, the induction of which inhibits the formation of FtsZ rings necessary for cell division (48). However, DNA damage is not the only cause of cell elongation in bacterial species such as E. coli. Exposure to antibiotics, such as ampicillin, can also result in cell elongation, albeit with a different nucleus-staining pattern when stained by DAPI (4′,6-diamidinophenylindole) (49). Ciprofloxacin treatment at bacteriostatic concentrations gives rise to distinct, multiple DAPI-stained nuclei in elongated cells (49, 50). In contrast, multiple DAPI-stained nuclei are generally not observed within the cytoplasm of single cells when E. coli is treated with bacteriostatic doses of ampicillin (49).

**FIG 7:**
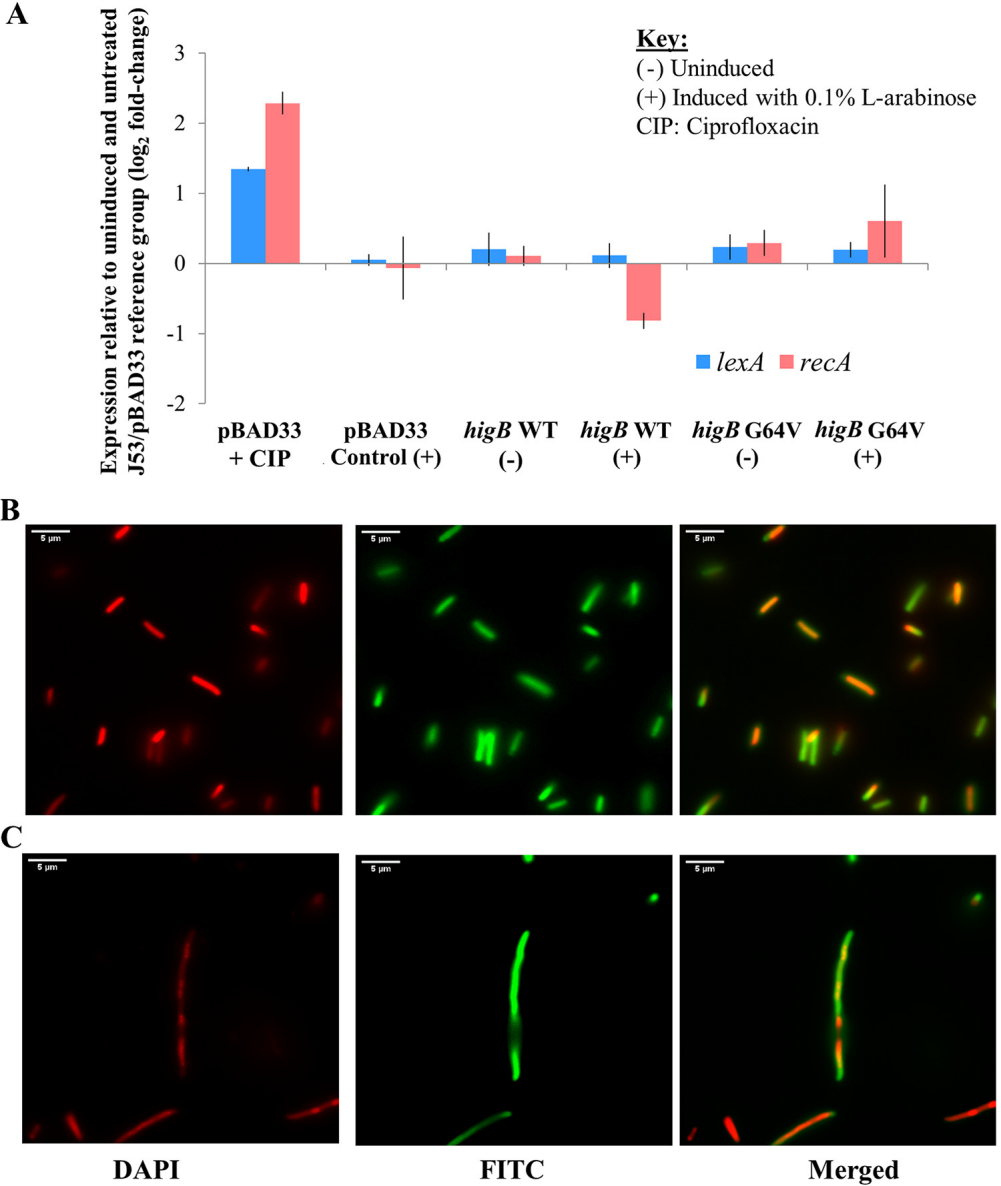
Overexpressing the IncC HigB toxin does not trigger the SOS response. (A) Whereas ciprofloxacin treatment upregulated *lexA* and *recA* in the J53/pBAD33 strain, which is a classic gene expression signature for the activation of the SOS response, overexpression of the wild-type *higB-*like toxin gene did not result in upregulation of *lexA* and *recA* relative to their expression in the untreated and uninduced J53/pBAD33 reference group. (B and C) Overexpression of the wild-type *higB-*like toxin gene resulted in a DAPI nucleus-staining pattern different from that of ciprofloxacin-treated J53-*gfpuv*. When *higB* is overexpressed in *trans*, DAPI staining can be observed throughout the cytoplasm (Fig. 8B). In ciprofloxacin-treated J53-*gfpuv*, DAPI staining revealed multiple distinct nuclei within the same elongated cells (Fig. 8C), which differs from the staining pattern observed in Fig. 8B.

We quantified levels of *lexA* and *recA* in the l-arabinose-induced J53-*gfpuv*/pBAD33-*higB*_v1 group relative to that in the uninduced empty vector control (J53-*gfpuv*/pBAD33) and found no evidence of *lexA* and *recA* upregulation in the *higB* overexpression group compared to their expression in the uninduced controls (J53-*gfpuv*/pBAD33 and uninduced J53-*gfpuv*/pBAD33-*higB*_v1) (Fig. 7A). Furthermore, fluorescence microscopy images from DAPI-stained J53-*gfpuv*/pBAD33-*higB*_v1 cells showed that multiple nuclei were not observed in the cytoplasm of elongated cells when *higB* expression was unopposed (Fig. 7B), unlike with the ciprofloxacin-treated J53-*gfpuv* control (Fig. 7C), in which distinct DAPI-stained nuclei are clearly visible within elongated cells. Taken together, our results imply that *higB* overexpression does not trigger the SOS response.

### Expression of the IncC *higBA* TA operon is activated by ciprofloxacin treatment.

In the *Alphaproteobacterium* Caulobacter crescentus, the chromosomal *higBA* operon is upregulated upon exposure to DNA-damaging compounds, such as the fluoroquinolone antibiotic ciprofloxacin (26). To examine whether this phenomenon is applicable to IncC *higBA*, we treated two J53-*gfpuv* strains that carry an IncC *higBA*-bearing plasmid (pACYC184-*higBA_*v1 or pEc158ΔMDR-*tetA*) with a sub-MIC of ciprofloxacin. pACYC184-*higBA_*v1 offers a simplified genetic context to quantify *higBA* expression under the control of its native promoter without confounding factors such as putative regulatory elements of neighboring operons in the IncC backbone. pEc158ΔMDR-*tetA* has the original backbone of the IncC clinical plasmid pEc158, but its multidrug resistance (MDR) region was replaced with a tetracycline resistance gene (*tetA*) to eliminate the potential effects of other resistance genes on the plasmid-bearing strain’s susceptibility to ciprofloxacin. A low ciprofloxacin concentration of 0.02 μg/ml was chosen to minimize cell death and potential alternations in plasmid copy numbers. The expression levels of *higB*, *higA*, *lexA*, and *recA* were quantified relative to those of the untreated reference group for each strain. For internal normalization of target gene expression levels, 16S rRNA, *rpoB*, and *ssrA* (the nontranslated tRNA-like domain) were collectively used as internal reference genes. *ssrA* was selected in addition to the frequently used 16S rRNA and *rpoB* gene, because *ssrA* had previously been identified as a robust internal reference for gene expression studies in which E. coli was treated with DNA-damaging agents (51).

**FIG 8:**
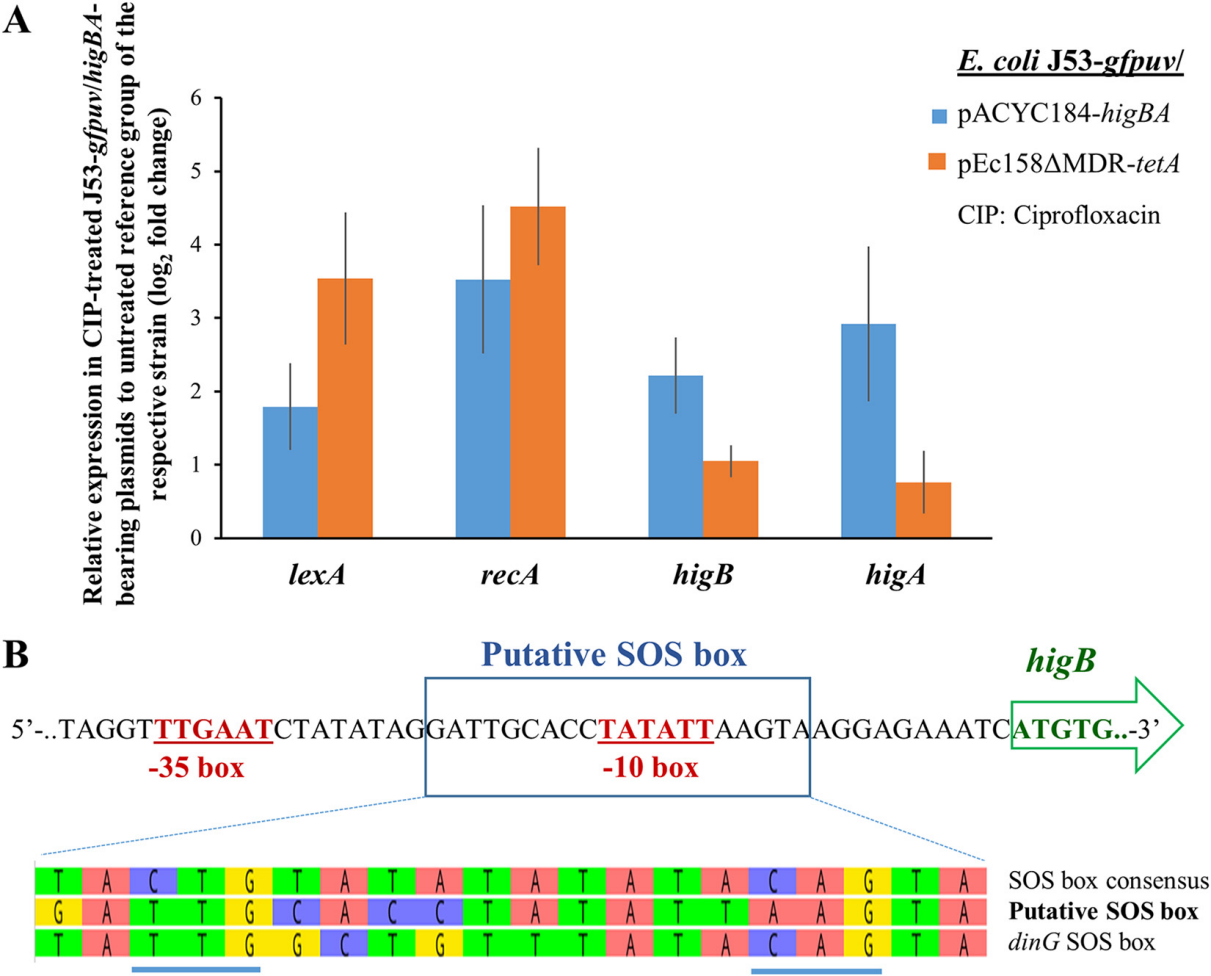
Expression of the *higBA-*like operon of IncC plasmids increases in response to ciprofloxacin treatment. (A) J53-*gfpuv* host strains with two different *higBA* operon-containing plasmids, pACYC184-*higBA_*v1 and pEc158ΔMDR-*tetA*, were treated with a sub-MIC of ciprofloxacin (0.02 μg/ml). An increase in the expression levels of *lexA*, *recA*, *higB*, and *higA* were observed in the ciprofloxacin-treated groups relative to those in the untreated reference groups for the same strains. (B) The promoter region of *higBA* of IncC plasmids contains a putative noncanonical SOS box that shares 65% and 60% homology with the canonical SOS box in E. coli and the SOS box of *dinG,* respectively. The positions of the two highly conserved trimers in the canonical sequence for SOS boxes in E. coli are indicated by the blue lines.

As expected, both *lexA* and *recA* were upregulated in the ciprofloxacin-treated pACYC184-*higBA_*v1 and pEc158ΔMDR-*tetA* groups relative to their expression in the untreated reference groups for the same strains (Fig. 8A), which was indicative of ciprofloxacin-induced DNA damage. The relative expression levels of *higB* showed a concomitant increase in both strains, with a higher upregulation (mean log_2_ fold change ± SEM = 2.22 ± 0.51, *n* = 3) in the J53-*gfpuv*/pACYC184-*higBA_*v1 strain than that in the J53-*gfpuv*/pEc158ΔMDR-*tetA* strain (mean log_2_ fold change ± SEM = 1.05 ± 0.22, *n* = 3). The expression level of the cognate antitoxin gene *higA* also increased in ciprofloxacin-treated samples.

To rule out the possibility that the increase in *higBA* expression levels was due to an increase in plasmid copy number in response to ciprofloxacin treatment, we quantified the change in plasmid copy number in the ciprofloxacin-treated group from that in the untreated reference group. In the J53-*gfpuv*/pACYC184-*higBA_*v1 strain, the addition of 0.02 μg/ml ciprofloxacin caused an average decrease of 20% in plasmid copy number relative to that in the untreated reference group (mean ± SEM = 0.80 ± 0.18, *n* = 4). In the J53-*gfpuv*/pEc158ΔMDR-*tetA* model, the relative plasmid copy number in the ciprofloxacin-treated group was 1.02 ± 0.36 (mean ± SEM, *n* = 4). These results clearly indicate that the expression of the IncC *higBA* TA system is upregulated by ciprofloxacin treatment.

In the noncoding region upstream of the IncC *higB* gene (Fig. 8B), a putative promoter with a −10 box (5′-TATATT-3′) and a −35 box (5′-TTGAAT-3′) were predicted by the BPROM bacterial promoter algorithm (52). This predicted promoter region (Fig. 8B) is identical upstream of both variants of *higB* (v1 and v2) in IncC plasmids and contains a putative, noncanonical SOS box. In *Gammaproteobacteria* such as E. coli, the consensus sequence of the canonical SOS box is 5′-TACTG(TA)_5_CAGTA-3′, with a pair of conserved trimers (underlined) separated by an AT-rich 10-bp spacer sequence (53–55). Although the trimers are highly conserved, noncanonical SOS boxes that contain variations in the first or final nucleotides in the trimer sequences have also been reported or computationally predicted (54–57). A notable example is the experimentally characterized SOS box upstream of *dinG* (5′-TATTGGCTGTTTATACAGTA-3′), which contains TTG instead of the usual CTG (58). This atypical trimer is also found in the left-hand side of the putative SOS box of IncC *higBA* (5′-GATTGCACCTATATTAAGTA-3′). The atypical AAG trimer on the right-hand side of the putative SOS box has also been found in the putative SOS boxes upstream of genes that are thought to be regulated by LexA (54, 57). Sequence alignment shows that the putative SOS box upstream of *higBA* shares 65% and 60% identity with the canonical sequence for E. coli SOS boxes and the *dinG* SOS box, respectively (Fig. 8B). Given the minor deviations in both sets of conserved trimers, potential interactions between LexA repressors and the putative SOS box are expected to be weak and need to be confirmed by further experiments, such as electrophoretic mobility shift assay and DNA footprinting.

## DISCUSSION

In this study, we characterized the *higBA* locus found in IncC plasmids and identified bioinformatic, structural, and functional similarities with other plasmid-mediated or chromosomal *higBA* TA systems reported in the literature (21–23, 25, 26, 28). We found that the expression of the IncC plasmid *higB*-like toxin gene in *trans* strongly inhibits the growth of E. coli, as has been reported for the expression of *higB* genes from the chromosomes of V. cholerae and M. tuberculosis and from the plasmids Rts1 (in P. vulgaris) and pAB120 (in A. baumannii) (21–23, 28). This type of postsegregational killing or growth inhibition plays a key role in maintaining plasmids or other genetic elements (16, 59), and we show that the IncC *higBA* TA system is highly effective in plasmid maintenance. This system is common in conjugative IncC plasmids in different bacterial species that are important carriers of multiple-drug resistance genes, including β-lactamase and carbapenemase genes, and thereby plays a major role in their spread and persistence.

The putative IncC plasmid antitoxin HigA significantly attenuates HigB toxicity in simple coexpression experiments, and it appears that the *higBA* pair is a type II TA system, with HigB belonging to the large RelE/ParE superfamily of endoribonucleases (such as RelE) and gyrase poisons (such as ParE) (60). HigB toxins have been defined as translation-dependent mRNases in Vibrio cholerae, Proteus vulgaris, and E. coli (21, 25, 28), and two different HigB toxins (from the V. cholerae chromosome and the Rts1 plasmid of P. vulgaris) inhibit protein synthesis through translation-dependent mRNA cleavage in a manner similar to, but distinct from, that of RelE (28). We show here that the IncC plasmid HigB-like toxin strongly depletes the transcript abundance of adenine-rich mRNA substrates, such as *lpp*, *opmA*, and the mRNA-like domain of the tmRNA *ssrA*, broadly in keeping with the observed effects of *higB* expression from V. cholerae, M. tuberculosis, and plasmid Rts1 on mRNA substrates in E. coli (21, 23, 28). More importantly, our results corroborate previous findings that HigB homologs deplete only translated mRNA substrates and exhibit negligible endonuclease activity against the same genes if they are not translated (23, 28). In other studies, this has been successfully demonstrated by altering the start codons (28, 61) or by measuring the transcript abundance of different regions of the same gene that contains translated and untranslated regions (e.g., the mRNA- and tRNA-like domains of the tmRNA *ssrA*). However, unlike *lpp* and *ompA* mRNAs, which contain numerous in-frame AAA codons that code for lysine, the AAA sequences in the mRNA-like domain of *ssrA* are all out of frame. This seems to suggest that the IncC plasmid toxin is capable of cleaving out-of-frame AAA sequences in translated RNA substrates in a way that is similar to that of the HigB toxin from the Rts1 plasmid (21). A single glycine at position 64 appears essential, as a G64V mutation completely abolished mRNA cleavage activity and growth inhibition by IncC HigB toxin. The critical role of highly conserved single amino acid residues for toxin activity was previously reported for a highly conserved histidine (H) residue, H92, in the Rts1 plasmid of P. vulgaris, where the H92Q mutation in Rts1 HigB abolished HigB-mediated growth arrest and mRNA cleavage (17). Similarly, three highly conserved amino acids in RelE (R61, R81, and Y87) are important for mRNA cleavage (62), while the conserved H87 residue in the RelE-type endoribonuclease YafQ is responsible for its toxicity and mRNA cleavage activity (63). In this study, we confirmed that the G64 residue of IncC HigB is highly conserved among HigB toxins from many other bacteria, although the position varies between the 56th and 65th residues among them.

While unopposed expression of the IncC *higB* gene resulted in an elongated cell morphology, there was no evidence to suggest that overexpressing *higB* can activate the SOS response. Our findings for the IncC *higBA* TA system, previously described as *tad/ata* (10, 13), are in keeping with the mechanism of action of RelE, which is probably the best understood of these bacterial endoribonuclease-type toxins to date. RelE has conserved basic residues that are critical for its function and undergoes only slight conformational change on the ribosomal binding for its mRNase activity (62). The role of RelE in increasing relative levels of charged tRNAs may be important in improving the fidelity of translation and overall translational control in stressful situations (62).

DNA damage induces several chromosomal TA systems in E. coli (*symER*, *hokE*, *yafN/yafO*, and *tisAB/istR*, whose promoters contain SOS boxes) (54, 64–68). In particular, the *tisAB/istR* system has been shown to contribute to SOS-induced drug tolerance and persister formation in E. coli (68). Here, we identified a putative, noncanonical LexA binding motif in the promoter region of the IncC TA system that shows minor deviations from the canonical SOS box (Fig. 8B). The *higBA* operon showed upregulation following ciprofloxacin treatment, mirroring previous observations of chromosomal *higBA* in C. crescentus (22). However, a direct role of the SOS response in regulating the expression of the IncC TA system remains to be experimentally confirmed, so the ciprofloxacin-induced upregulation of *higBA* may be due either to the direct effects of DNA damage or to the pleiotropic effects of ciprofloxacin.

In the HigBA TA system of A. baumannii plasmid pAB120, which shares the highest homology with the TA system of the IncC plasmid (Fig. 1), the antitoxin (HigA2_Ab, where “Ab” stands for A. baumannii) and toxin-antitoxin (HigBA2_Ab) complexes are thought to repress the transcription of the *higBA* operon (22). It has recently been suggested that LexA often represses the expression of genes carried by mobile genetic elements (MGEs) in concert with a transcription factor that can be encoded by the MGE itself (69). Some MGE genes that are regulated in this way have noncanonical SOS boxes in their promoters (69–71). If the antitoxin and toxin-antitoxin encoded by the IncC plasmids can also repress the *higBA* operon, our observation of a putative, noncanonical SOS box upstream of the IncC plasmid TA system raises the possibility that this mobile plasmid addiction module is jointly regulated by the SOS response and the antitoxin/toxin-antitoxin complexes.

## MATERIALS AND METHODS

### Bioinformatics.

Phylogenetic trees were constructed using amino acid sequences of HigB/RelE toxins and HigA/RelB antitoxins from 7 bacterial species and those of their homologs found in IncC plasmids using MEGA X (72). The evolutionary history was inferred using the maximum-likelihood method. Initial trees for the heuristic search were obtained automatically by applying the neighbor-joining and BioNJ algorithms to a matrix of pairwise distances estimated using a JTT model and then by selecting the topology with the superior log likelihood value and with a bootstrap value of 500.

The alignment of amino acid sequences was performed using ClustalW of MEGA X. The secondary protein structures of toxin and antitoxin were predicted using the PSIPRED 4.0 tool with default settings (73). Modeling of protein tertiary structures was performed using the SWISS-MODEL tool (74). DNA sequence alignments were performed using the Geneious software (Biomatters, New Zealand).

BLASTN was used to determine the distribution of the TA system associated with IncC plasmids in the GenBank database. The combined nucleotide sequence (658 bp) for the predicted toxin and antitoxin genes in an IncC plasmid of clinical origin (pEc158) was used as the input. For this analysis, megaBLAST was performed by using the following parameters: (i) an expectation threshold (E value) of ≤0.01 and a score greater than 40, (ii) a maximum number of target sequences of 1,000, (iii) automatically adjusted parameters for short input sequences, and (iv) different match/mismatch scores to identify high- to low-conservation sequences. Separate BLASTP searches were performed using the amino acid sequences of the toxin and antitoxin of IncC plasmids as separate inputs against a nonredundant protein sequence database and reference protein sequence database, with E values less than or equal to 0.01 and with coverage of ≥80%.

### Construction of bacterial strains.

The oligonucleotide primers (see Table S5 in the supplemental material), plasmids (Table S6), and bacterial strains (Table S7) used in this study are shown in the supplemental material. The sodium azide-resistant E. coli reference strain J53 was used as the host strain for all growth rate assays. J53 was chromosomally tagged with a green fluorescence gene (*gfpuv*) driven by a strong promoter (P_T7A1_) to generate the J53-*gfpuv* strain, which was used in plasmid stability assays and fluorescence microscopy experiments. Both strains were used for quantitative real-time PCR (qRT-PCR) assays.

10.1128/mSphere.00424-21.9 TABLE S6 Plasmids used in this study. Download Table S6, DOCX file, 0.01 MB. Copyright © 2021 Qi et al. 2021 Qi et al. https://creativecommons.org/licenses/by/4.0/ This content is distributed under the terms of the Creative Commons Attribution 4.0 International license.

10.1128/mSphere.00424-21.10 TABLE S7 Bacterial strains used in this study. Download Table S7, DOCX file, 0.01 MB. Copyright © 2021 Qi et al. 2021 Qi et al. https://creativecommons.org/licenses/by/4.0/ This content is distributed under the terms of the Creative Commons Attribution 4.0 International license.

Using a CRISPR-based method (75), P_T7A1_-*gfpuv* was inserted into E. coli J53 at the SS9 chromosomal site between *aslA* and *glmZ*. Briefly, J53 was transformed with two helper plasmids, pX2-Cas9 (75) and pKM200 (76), which encode an l-arabinose-inducible *cas9* gene and the lambda red recombinase system, respectively. The P_T7A1_-*gfpuv* fragment, flanked by homologous regions to the SS9 insertion site, was PCR amplified from the pSS9 plasmid template using the primer pair SS9_*gfpuv*-F and -R. The PCR product and the SS9_RNA plasmid that carried the guide RNA (75) were simultaneously electroporated into the J53/pKM200/pX2-Cas9 strain, which was then grown immediately in SOC medium (2% tryptone, 0.5% yeast extract, 10 mM NaCl, 2.5 mM KCl, 10 mM MgCl_2_, 10 mM MgSO_4_, and 20 mM glucose in sterile water) at 37°C for 4 h. Cas9 expression was induced by adding 0.4% l-arabinose to the SOC medium. The J53-*gfpuv* insertion mutant was selected on ampicillin-, kanamycin-, and l-arabinose-containing LB agar. Subsequently, the pKM200 and SS9_RNA plasmids were cured by passaging the J53-*gfpuv* strain in antibiotic-free LB medium for 4 days at 37°C.

The 38.2-kb multidrug resistance (MDR) region in the IncC plasmid pEc158 was deleted by homologous recombineering. Primers pEc158ΔMDR-*tetA-*F1 and pEc158ΔMDR-*tetA-*R1 were used to amplify the *tetA* resistance gene marker from pACYC184 and introduce 40-bp overhang sequences that are homologous to the recombineering sites flanking the MDR. The overhang sequences were further extended to 80 bp by PCR amplification with the PCR product with primers pEc158ΔMDR-*tetA-*F2 and pEc158ΔMDR-*tetA-*R2. The resulting PCR product was electroporated into the J53-*gfpuv*/pEc158/pKM200 strain. After 4 h of recovery in SOC medium at 37°C, the J53-*gfpuv*/pEc158ΔMDR-*tetA* mutant was isolated on LB agar that contains tetracycline (10 μg/ml).

### Growth rate assays.

The coding regions of the two variants of the IncC toxin *higB* gene, v1 and v2, were PCR amplified with the primers *higB*_EcoRI-F and *higB*_HindIII-R, using the IncC plasmids pEc158 (39) and JIE1709 (77) as the templates, respectively. The PCR products were cloned into the pBAD33 vector at the EcoRI/HindIII position of the multiple-cloning site (78). The IncC antitoxin *higA* gene PCR amplified with the *higA*_EcoRI-F and *higA*_HindIII-R primers was cloned into the pBAD33 and pBAD24 vectors at the same EcoRI/HindIII site (78). The E. coli J53 strain was transformed with pBAD33, pBAD33-*higB*_v1, pBAD33-*higB*_v2, and pBAD33-*higA*. Overnight cultures of the four above-mentioned strains were adjusted to an OD at 600 nm (OD_600_) of approximately 1 and diluted 250-fold in fresh gentamicin (8 μg/ml)-containing LB Lennox medium at the start of the growth experiment with and without 0.04% l-arabinose induction. Diluted bacterial cultures were transferred in triplicates of 200-μl aliquots to 96-well microplates (Corning, USA) and incubated with shaking at 37°C overnight in a SpectraMax iD5 multi-mode microplate reader (Molecular Devices, USA). The OD_600_ was measured every 10 min for 10 h.

For the growth rescue experiment using induced antitoxin expression, J53/pBAD33-*higB*_v1 was transformed with the pBAD24 and pBAD24-*higA* plasmids. The J53/pBAD33/pBAD24 control strain and the J53/pBAD33-*higB*_v1/pBAD24 and J53/pBAD33-*higB*_v1/pBAD24-*higA* experimental groups were grown in LB medium that contained both gentamicin and ampicillin with and without l-arabinose induction.

### Plasmid stability assay by FACS.

Variant 1 of the IncC toxin-antitoxin system was PCR amplified with its putative 94-bp promoter region using primers *higBA*_TAS_v1_XbaI_F and *higBA_*TAS_v1_BamHI-R (where “TAS” stands for the TA system), with the pEc158 plasmid as a template (39). Similarly, variant 2 of the IncC TAS was PCR amplified with its 94-bp promoter region using primers *higBA*_TAS_v2_XbaI_F and *higBA*_TAS_v2_BamHI-R, with the JIE1709 plasmid as a template (77). The PCR products were subcloned into pGEM-T-Easy (Promega, USA) before being cloned into the pACYC184 vector at the XbaI/BamHI site. Cold CaCl_2_-treated J53-*gfpuv* competent cells were transformed with pACYC184, pACYC184-*higBA*-v1, and -v2 plasmids by heat shock.

Prior to each plasmid stability assay, J53-*gfpuv* with the pACYC184 control plasmid, pACYC184-*higBA*-v1, and pACYC184-*higBA*-v2 were streaked on LB agar containing 25 μg/ml chloramphenicol and incubated at 37°C overnight. A single colony of each strain was picked to inoculate 10 ml of LB medium in 50-ml Falcon tubes. After overnight growth at 37°C with continuous shaking (225 rpm), 10 μl of overnight culture was diluted in 10 ml of fresh LB medium the next morning, which was cultured under the same conditions for 8 h. This cycle of overnight (16-h) and daytime (8-h) incubation was carried out continuously for 4 days (96 h) in three different weeks to obtain 3 biological replicates.

One hundred microliters of saturated cultures by the end of each 8- or 16-h incubation period was diluted in 900 μl of sterile phosphate-buffered saline (PBS) medium (Bio-Rad, USA) and centrifuged for 3 min at 9,000 rpm. The pellet was resuspended in 500 μl PBS, which was further diluted 1:20 in PBS for FACS. FACS of single bacterial cells was performed on the Influx 5 laser sorter (BD Biosciences) using the 140-μm nozzle. The flow sheath was filtered with a 0.1-μm in-line filter. Green fluorescence was detected with the 488-nm laser with the 530/40-bandpass filter.

Bacterial cells were sorted into individual wells of a round-bottom 96-well microtiter plate containing 200 μl LB medium without antibiotics. After overnight growth at 37°C, 2 μl of each viable culture was diluted 200-fold in LB containing 20 μg/ml chloramphenicol and incubated overnight at 37°C. On the next day, the proportion of viable cultures that grew in 20 μg/ml chloramphenicol was recorded.

Experimental validation was performed to verify the stringency of the FACS procedure in sorting individual cells into individual wells of 96-well microtiter plates. Immediately after cell sorting, the contents of 27 randomly selected wells were plated on individual antibiotic-free LB agar plates and incubated at 37°C. Single colonies were observed on 93.4% of the plates after overnight incubation, while the remaining plates contained no colonies. No plates contained more than one colony.

### Isolation of escape mutants.

The J53/pBAD33-*higB*_v1 ancestral strain was grown with l-arabinose induction for 16 h under the same incubation conditions as used in the growth rate assays described previously. This procedure was carried out on three different days, with 9 replicates included on each day, for a total of 27 independent cultures. The endpoint OD_600_ for each culture was measured on the SpectraMax iD5 multi-mode microplate reader (Molecular Devices, USA). Cultures containing escape mutant isolates are defined as those with an endpoint blank-corrected OD_600_ of at least 0.4. The 9 endpoint cultures that met the criteria were streaked on LB agar containing 8 μg/ml gentamicin and incubated overnight at 37°C. Nine individual colonies of escape mutants were isolated and grown overnight before glycerol stocks were prepared for each strain. Plasmid samples were extracted from each escape mutant strain using the QIAprep minispin kit (Qiagen, Germany) for Sanger sequencing with primers pBAD_Seq_F and pBAD_Seq_R.

### Quantitative real-time PCR.

Stationary-phase cultures of E. coli J53 bearing the pBAD33, pBAD33-*higB*_v1 (wild-type), and pBAD33-*higB*_v1 (G64V) plasmids were diluted 1:50 in 4 ml gentamicin-containing (8 μg/ml) LB medium and incubated with shaking at 37°C. After approximately 90 min, each culture was divided into 2-ml cultures, one with 0.1% l-arabinose induction and one without. A separate J53/pBAD33 culture was treated with 0.1 μg/ml ciprofloxacin. The induced and uninduced cultures were grown for 2 to 3 h and harvested by centrifugation at 10,000 rpm for 3 min.

In 3 biological replicates, late-exponential-phase cultures of J53-*gfpuv*/pACYC184-*higBA_*v1 with an OD_600_ of approximately 0.5 were diluted 1:30 in LB medium–20 μg/ml chloramphenicol with or without ciprofloxacin (0.02 μg/ml). Similarly, cultures of J53-*gfpuv*/pEc158ΔMDR-*tetA* were diluted in LB medium containing tetracycline (10 μg/ml) under the same experimental conditions. After 2 to 3 h, bacterial pellets were harvested by centrifugation.

Bacterial pellets were treated with 400 μg/ml lysozyme in Tris-EDTA (TE) buffer. Total RNA was purified using the NucleoSpin RNA Plus kit (Macherey-Nagel, Germany) according to the manufacturer’s instructions. The procedure was repeated to obtain 3 biological replicates of total RNA. Three micrograms of each sample was treated with a Turbo DNA-free kit (Invitrogen, USA) to eliminate genomic DNA. Reverse transcription was carried out using 100 ng of each RNA sample as a template using a high-capacity cDNA reverse transcription kit (Applied Biosystems, USA) according to the manufacturer’s instructions.

All cDNA samples were amplified using gene-specific qRT-PCR primers (Table S5) and SYBR green PCR reagent (Qiagen, Germany) on a Rotor-Gene 6000 real-time thermocycler. The thermocycler profile included 5 min of initial denaturation at 95°C and 45 cycles of denaturation (95°C for 5 s) and annealing/extension (60°C for 10 s). Reactions were carried out in 2 technical replicates for 3 biological replicates. The threshold cycle (CT) values of all reactions were obtained using the Rotor-Gene Q series software. The internal reference genes were 16S rRNA, *rpoB*, and *gyrB* for all qRT-PCR assays, except with the experimental sets with ciprofloxacin-treated bacterial sample groups, for which *gyrB* was replaced by the tRNA-like domain of *ssrA.* Using the 2^–ΔΔCT^ method, the relative expression levels of the target genes were normalized to the average expression of the three internal reference genes. The batch effect was accounted for by normalizing the relative expression levels in the experimental groups to those in the reference groups within each batch of biological replicates. Relative expression was calculated as a log_2_ fold change.

Plasmid copy number variations in ciprofloxacin-treated cultures (J53-*gfpuv*/pACYC184-*higBA*_v1 and pEc158ΔMDR-*tetA*) relative to copy numbers of the untreated reference groups were quantified using a qRT-PCR method described by Lee et al. (79). Genomic and plasmid DNA were coextracted from the two strains grown under the same conditions as described for RNA extraction with and without 0.02 μg/ml ciprofloxacin using the QIAamp DNA minikit (Qiagen, Germany) in 4 biological replicates. *higB* and *rpoB* were used as single-copy genes in plasmid DNA and chromosomal DNA, respectively. The 2^–ΔΔCT^ method was applied to calculate the plasmid DNA copy number in the ciprofloxacin-treated groups relative to those in the untreated reference groups.

### Fluorescence microscopy.

Stationary-phase cultures of green fluorescence protein-tagged E. coli J53-*gfpuv*, as well as E. coli J53-*gfpuv* containing pBAD33-*higB*_v1 (wild-type) and pBAD33-*higB*_v1 (G64V), were diluted 1:50 in 4 ml fresh LB medium (with 8 μg/ml gentamicin for plasmid-bearing strains) and incubated with shaking at 37°C. After approximately 90 min, each culture was divided into two sets of 2-ml cultures, with one set designated the uninduced and nontreated control group. In the treated or induced set, E. coli J53-*gfpuv* was treated with 0.1 μg/ml ciprofloxacin, while E. coli J53-*gfpuv*/pBAD33-*higB*_v1 (wild-type or G64V) was induced with 0.1% l-arabinose. After 3 h of incubation, bacterial cells were pelleted, washed, and resuspended in sterile PBS. DAPI staining was performed for 15 min at a concentration of 2 μg/ml. Five microliters of each sample was placed between a SuperFrost Ultra Plus adhesion slide (ThermoFisher Scientific, USA) and a coverslip.

Fluorescence microscopy images were acquired using the DeltaVision Elite deconvolution microscope (GE Life Sciences, USA). Green fluorescence and blue fluorescence signals were obtained on the fluorescein isothiocyanate (FITC) and DAPI channels, respectively. The 100× lens objective was used, and augmented magnification was applied to achieve a magnification factor of ×160. The exposure times were 0.8 s, 0.2 s, and 1.0 s for FITC, DAPI, and plane-polarized light, respectively. Image processing and analysis were performed using the ImageJ software (National Institutes of Health, USA). Average cell lengths were determined by drawing straight lines along the lengths of 5 randomly selected E. coli cells in Fig. 6A to D. The lengths of the cells were calculated using the 5 μM scale bar in each figure as a calibration gauge.

### Statistics.

Welch’s two-sample *t* test for unequal variances was performed using the t.test function in R. The *P* values for the *t* test results are summarized as *P* values of <0.05 (*), <0.01 (**), and <0.001 (***). Unless otherwise stated, all measured quantities in this study are calculated from biological replicates and expressed as means ± SEMs (standard errors of the means). All error bars in the figures represent SEMs.
