# Supplementary figures and images for: The higBA-Type Toxin-Antitoxin System in IncC Plasmids Is a Mobilizable Ciprofloxacin-Inducible System
Source: mSphere. 2021 Jun 2;6(3):e00424-21. doi: 10.1128/mSphere.00424-21 (PMC8265657; doi:10.1128/mSphere.00424-21)

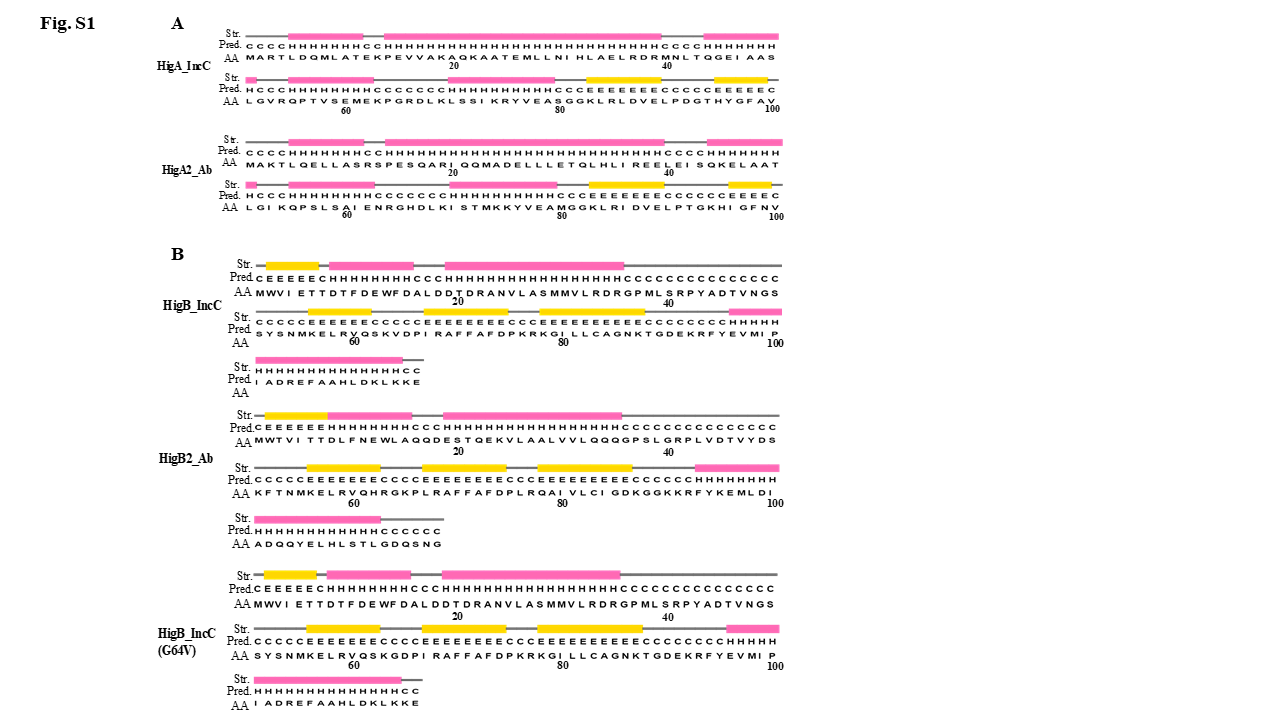

Supplement: FIG S1 [file msphere.00424-21-sf001.tif]

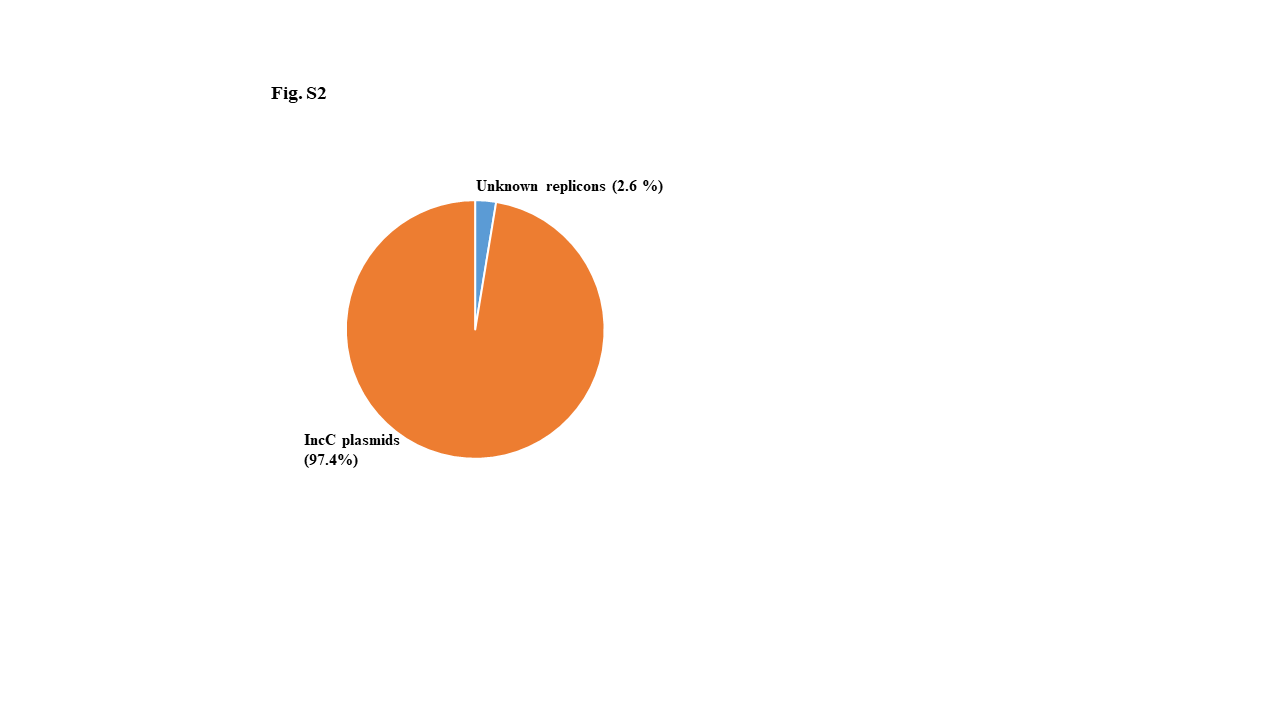

Supplement: FIG S2 [file msphere.00424-21-sf002.tif]

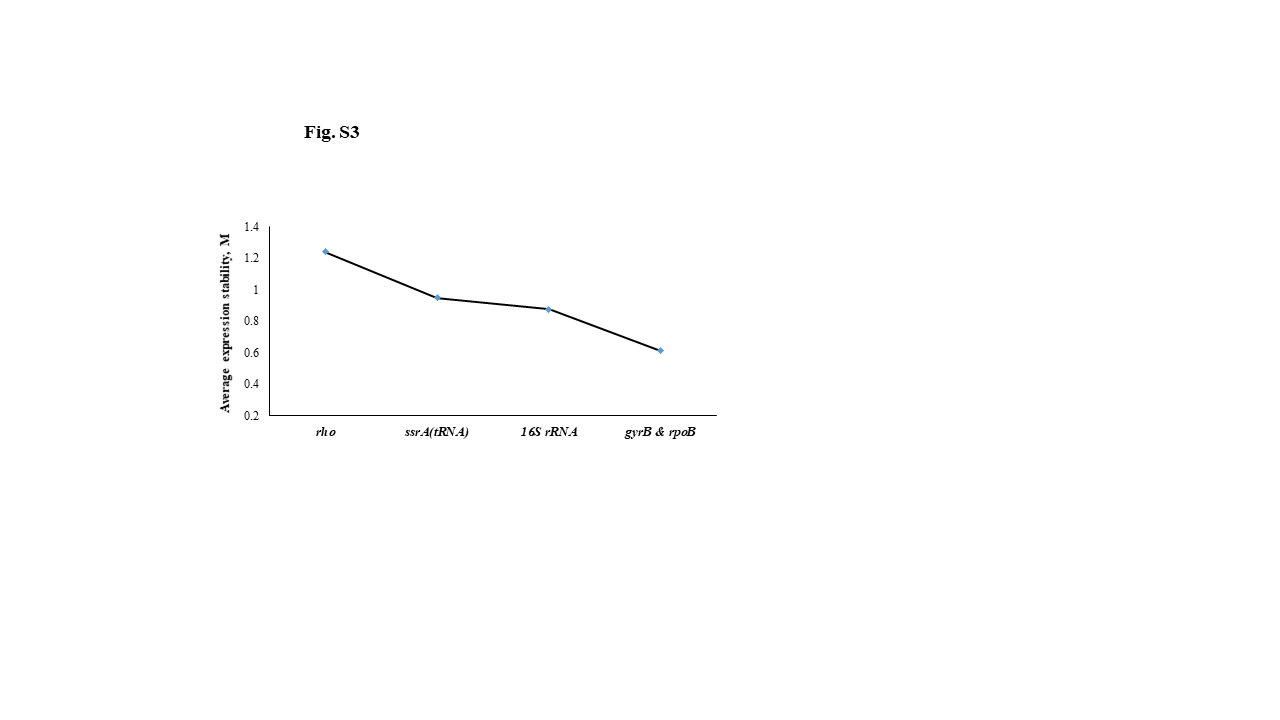

Supplement: FIG S3 [file msphere.00424-21-sf003.tif]
