# Supplementary material for: The higBA-Type Toxin-Antitoxin System in IncC Plasmids Is a Mobilizable Ciprofloxacin-Inducible System
Source: mSphere. 2021 Jun 2;6(3):e00424-21. doi: 10.1128/mSphere.00424-21 (PMC8265657; doi:10.1128/mSphere.00424-21)
Supplement: TABLE S2 [file msphere.00424-21-st002.docx]

**Table S2**

| **Amino acid position** | **Amino acid** | **Codon** | |
| --- | --- | --- | --- |
|  |  | **Variant 1 (v1)** | **Variant 2 (v2)** |
| 7 | T | ACC (0.47) | ACT (0.16) |
| 16 | A | GCT (0.11) | GCA (0.21) |
| 23 | A | GCA (0.21) | GCC (0.31) |
| 36 | G | GGC (0.46) | GGT (0.29) |
| 41 | R | AGG (0.03) | AGA (0.02) |
| 58 | L | CTT (0.12) | CTG (0.46) |
| 68 | R | AGA (0.02) | AGG (0.03) |
| 69 | A | GCG (0.38) | GCA (0.21) |
| 104 | R | CGT (0.36) | CGC (0.44) |

The ratios shown in parentheses represent the abundance of codon relative to all codons that code for the same amino acid in the *E. coli* K12 wild-type strain – the ancestral strain for *E. coli* J53 (1).

Reference

1. Nakamura Y, Gojobori T, Ikemura T. Codon usage tabulated from international DNA sequence databases: Status for the year 2000. Nucleic Acids Res. 2000;28(1):292.
