# Supplementary material for: The higBA-Type Toxin-Antitoxin System in IncC Plasmids Is a Mobilizable Ciprofloxacin-Inducible System
Source: mSphere. 2021 Jun 2;6(3):e00424-21. doi: 10.1128/mSphere.00424-21 (PMC8265657; doi:10.1128/mSphere.00424-21)
Supplement: TABLE S4 [file msphere.00424-21-st004.docx]

**Table S4**

| **Isolate ID** | **Type of mutation** | **Site of mutation** | **Descriptions** |
| --- | --- | --- | --- |
| 1 | Insertion | *higB* coding region | Insertion of IS4-like element ISVsa5 family transposase from the chromosome of the *E. coli* J53 host strain |
| 2 | Deletion | Starting from base pair position 38 in the *higB* coding region | 14 bp (5'-GTTCGATGCTCTGG-'3') frameshift deletion |
| 3 | Insertion | *higB* coding region | Insertion of IS4-like element ISVsa5 family transposase from the chromosome of the *E. coli* J53 host strain |
| 4 | Insertion | *higB* coding region | Insertion of IS3-like element IS2 family transposase from the chromosome of the *E. coli* J53 host strain |
| 5 | Point mutation | Base pair position 191 in the *higB* coding region | Nucleotide substitution G191T resulting in the replacement of a glycine residue with valine at the 64^th^ amino acid position (G64V) of HigB protein |
| 6 | Insertion | Between base pair positions 201 and 202 in the *higB* coding region | In-frame insertion of a CCC codon, resulting in insertion of a proline residue between amino acid positions 67 and 68 |
| 7 | Insertion | L-arabinose inducible promoter | Insertion of IS1-like element IS1A family transposase from the chromosome of the *E. coli* J53 host strain |

Seven of the nine escape mutants evolved from *E. coli* J53 + pBAD33-*higB_*v1 in this work were successfully sequenced by Sanger sequencing.
