# Supplementary material for: The higBA-Type Toxin-Antitoxin System in IncC Plasmids Is a Mobilizable Ciprofloxacin-Inducible System
Source: mSphere. 2021 Jun 2;6(3):e00424-21. doi: 10.1128/mSphere.00424-21 (PMC8265657; doi:10.1128/mSphere.00424-21)
Supplement: TABLE S5 [file msphere.00424-21-st005.docx]

**Table S5**

| **Primer name** | **DNA oligonucleotide sequence (5’->3’)** | **PCR/qRT-PCR product** | **Source** |
| --- | --- | --- | --- |
| *higB*_EcoRI-F | GCGAATTCATGTGGGTCATCGAGA | *higB*-like toxin gene v1 and v2 coding regions for cloning into pBAD33 | This study |
| *higB*_HindIII-R | GCAAGCTTTCACTCCTTCTTCAATTTATCCAAGT |  |  |
| *higA*_EcoRI-F | GCGAATTCATGGCAAGAACTCTTGA | *higA*-like antitoxin gene coding regions for cloning into pBAD33 and pBAD24 |  |
| *higA*_HindIII-R | GCAAGCTTGGCGTATCGAGTTAA |  |  |
| SS9_*gfpuv*-F | CCATCTAGACACCGCCACGCCGCCACCGTT | Green fluorescence gene *gfpuv* and flanking regions homologous to SS9 chromosomal insertion site for homologous recombineering |  |
| SS9_*gfpuv*-R | GTTGCAGAATAACCCGCCACA |  |  |
| *higBA_*TAS_v1_XbaI-F | TCTAGACTTACTGACATTTCGGCCC | *higBA*-like toxin-antitoxin system (TAS) with predicted promoter and toxin variant 1 for cloning into pACYC184 |  |
| *higBA_*TAS_v1_BamHI-R | GGATCCACGGCTGGAAGAAGA |  |  |
| *higBA_*TAS_v2_XbaI-F | TCTAGACTTACCGGCATTTCGTCCCC | *higBA-*like TAS with predicted promoter and toxin variant 2 for cloning into pACYC184 |  |
| *higBA_*TAS_v2_BamHI-R | GGATCCACGGCTGGAGGAAGTGCAAGA |  |  |
| pEc158ΔMDR-*tetA-*F1 | GCACCCCCTTGAGCTTTGCTCAGTCAGGACAGACCTATCA**AAGTTGTAATTCTCATG** | Tetracycline resistance gene *tetA* in pACYC184 with 80 bp homologous flanking regions for deleting the 38.2 kB MDR in pEc158 |  |
| pEc158ΔMDR-*tetA-*R1 | GATGGATTGCGCCCTTATTTGCTTTTGTGATGCCGGTGAA**AGGTGCCGCCGGCTTCC** |  |  |
| pEc158ΔMDR-*tetA-*F2 | GCCACTGGAGACGTGCTGGCGGAGTATATCTGGCTCGATG**GCACCCCCTTGAGCTTTGCT** |  |  |
| pEc158ΔMDR-*tetA-*R2 | CCTTTGCCAGGTTGCTACGCATTCTTGCCTTGACCGAAAG**GATGGATTGCGCCCTTATTT** |  |  |
| pBAD_Seq_F | CTATGCCATAGCATTTTTATCCATA | Primers for Sanger sequencing of the multiple-cloning site in pBAD33 and pBAD24 |  |
| pBAD_Seq_R | GTTCTGATTTAATCTGTATCAGGCT |  |  |
| *ssrA*-F1-RT | GGGTTGAAGCGTTAAAACTT | tRNA-like domain of *ssrA* tmRNA; internal reference gene for qRT-PCR |  |
| *ssrA*-R1-RT | TCCTCGGTACTACATGCTTA |  |  |
| *ssrA*-F2-RT | ATTTGCGAAACCCAAGGTGC | *ssrA* fragment spanning the mRNA-like domain; target gene for qRT-PCR |  |
| *ssrA*-R2-RT | TGACCTCTCTTGATCCCCGT |  |  |
| **Primer name** | **DNA oligonucleotide sequence (5’->3’)** | **PCR/qRT-PCR product** | **Source** |
| *lpp*-F-RT | ACTAAACTGGTACTGGGCGC | Major outer membrane lipoprotein gene (*lpp*); target gene for qRT-PCR | This study |
| *lpp*-R-RT | GCTGCGTCATCTTTAGCAGC |  |  |
| *higB*-F-RT | TACAGCAACATGAAAGAGCT | *higB*-like toxin gene; target gene for qRT-PCR |  |
| *higB*-R-RT | TTCAATTTATCCAAGTGCGC |  |  |
| *gyrB*_F_RT | ACCTGTTCGAGCCGATTGTT | Gyrase subunit B gene; internal reference gene for qRT-PCR |  |
| *gyrB*_R_RT | GCTCGCCACGTTCGATAAAC |  |  |
| *rho*_F_RT | GGTTGTAGTCGATAGCCGGG | Gene encoding Rho-factor for Rho-dependent termination; candidate internal reference gene for qRT-PCR |  |
| *rho*_R_RT | AAAGTGTTGACCGGTGGTGT |  |  |
| *higA*-F-RT | GAACTCTTGACCAAATGCTG | *higA*-like antitoxin gene; target gene for qRT-PCR |  |
| *higA*-R-RT | GCCACTGAGATGTTACTGAA |  |  |
| *rpoB*-F-RT | GTAAGGCACAGTTCGGTGGT | RNA polymerase subunit β gene (*rpoB*); internal reference gene for qRT-PCR | (2) |
| *rpoB*-R-RT | ATTTCCTGCAGGGTGTATGC |  |  |
| *lexA*-F-RT | CGCGGCTGAAGAACATCTGA | *lexA* gene; target gene for qRT-PCR | (3) |
| *lexA* -R-RT | GCGGCAACCCTTCTTCCTCT |  |  |
| *recA*-F-RT | GTTCCATGGATGTGGAAACC | *recA* gene; target gene for qRT-PCR | (4) |
| *recA*-R-RT | ATATCGACGCCCAGTTTACG |  |  |
| 16S rRNA-F-RT | GTTAATACCTTTGCTCATTGA | 16S ribosomal RNA gene; internal reference gene for qRT-PCR | (5) |
| 16S rRNA-R-RT | ACCAGGGTATCTAATCCTGTT |  |  |
| *ompA*-F-RT | CTGGTGCTAAACTGGGCTG | Outer membrane protein precursor A gene (*ompA*); target gene for qRT-PCR | (6) |
| *ompA*-R-RT | TTAACCTGGTAACCACCAAAAG |  |  |

Abbreviations in this table include *tetA*: tetracycline resistance gene; MDR: multi-drug resistance region.

**References**

2. Kamruzzaman M, Patterson JD, Shoma S, Ginn AN, Partridge SR, Iredell JR. Relative strengths of promoters provided by common mobile genetic elements associated with resistance gene expression in Gram-negative bacteria. Antimicrob Agents Chemother. 2015;59(8):5088-91.

3. Kamruzzaman M, Iredell J. A ParDE-family toxin antitoxin system in major resistance plasmids of Enterobacteriaceae confers antibiotic and heat tolerance. Sci Rep. 2019;9(1):9872.

4. Shilpakala SR, Raghunathan M. Impact of DNA gyrase inhibition by antisense ribozymes on *recA* in *E. coli.* Mol Biol Rep. 2009;36(7):1937-42.

5. Gao W, Zhang W, Meldrum DR. RT-qPCR based quantitative analysis of gene expression in single bacterial cells. J Microbiol Methods. 2011;85(3):221-7.

6. Camprubí-Font C, Ruiz Del Castillo B, Barrabés S, Martínez-Martínez L, Martinez-Medina M. Amino acid substitutions and differential gene expression of outer membrane proteins in adherent-invasive *Escherichia coli*. Front Microbiol. 2019;10:1707.
