# Supplementary material for: The higBA-Type Toxin-Antitoxin System in IncC Plasmids Is a Mobilizable Ciprofloxacin-Inducible System
Source: mSphere. 2021 Jun 2;6(3):e00424-21. doi: 10.1128/mSphere.00424-21 (PMC8265657; doi:10.1128/mSphere.00424-21)
Supplement: TABLE S6 [file msphere.00424-21-st006.docx]

**Table S6**

| **Plasmid name** | **Characteristics** | **Reference** |
| --- | --- | --- |
| pBAD33-Gm | Referred to as pBAD33 throughout this work; expression vector with p15A origin of replication and L-arabinose inducible promoter upstream of the multiple-cloning site; Gm^R^ | (7) |
| pBAD33*-higB*_v1  (pJIQQ30) | pBAD33 with variant 1 of the IncC *higB*-like toxin gene cloned into the EcoRI/HindIII site; Gm^R^ | This study |
| pBAD33-*higB*_v2  (pJIQQ31) | pBAD33 with variant 2 of the IncC *higB*-like toxin gene cloned into the EcoRI/HindIII site; Gm^R^ |  |
| pBAD33*-higA*  (pJIQQ32) | pBAD33 with the IncC *higA-*like antitoxin gene cloned into the EcoRI/HindIII sites; Gm^R^ |  |
| pBAD24 | Expression vector with pBR332 origin of replication and L-arabinose inducible promoter upstream of the multiple-cloning site; Amp^R^ | (7) |
| pBAD24-*higA*  (pJIQQ33) | pBAD24 with the IncC antitoxin gene cloned into the EcoRI/HindIII site; Amp^R^ | This study |
| pACYC184 | Expression vector with p15A origin of replication; Tc^R^ and Cm^R^ | (8) |
| pACYC184-*higBA*_v1  (pJIQQ34) | pACYC184 with the IncC toxin(v1)-antitoxin system and the promoter region cloned into the XbaI/BamHI site; Cm^R^ | This study |
| pACYC184-*higBA*_v2  (pJIQQ35) | pACYC184 with the IncC toxin(v2)-antitoxin system and the promoter region cloned into the XbaI/BamHI site; Cm^R^ |  |
| pEc158ΔMDR-*tetA* | IncC plasmid of clinical origin pEc158 with its multi-drug resistance (MDR) region replaced with tetracycline resistance gene *tetA*; Tc^R^ |  |
| pSS9 | Template plasmid with the *gfpuv* gene downstream of T7A1 promoter, flanked by homologous regions to the *E. coli* SS9 chromosomal insertion site between *glmZ* and *aslA*; Tc^R^ | (9) |
| SS9_RNA | Plasmid that expresses a guide RNA that targets the *E. coli* SS9 chromosomal insertion site; Amp^R^ |  |
| pX2-Cas9 | Plasmid with an L-arabinose inducible *cas9* gene; Kan^R^ |  |
| pKM200 | Temperature-sensitive helper plasmid that expresses genes of the lambda red recombinase system; Cm^R^ | (10) |

Abbreviations in this table for antibiotic resistance phenotypes include Gm^R^: gentamicin; Amp^R^: ampicillin; Cm^R^: chloramphenicol; Tc^R^: tetracycline; Kan^R^: kanamycin

**References**

7. Guzman LM, Belin D, Carson MJ, Beckwith J. Tight regulation, modulation, and high-level expression by vectors containing the arabinose P_BAD_ promoter. J Bacteriol. 1995;177(14):4121-30.

8. Chang AC, Cohen SN. Construction and characterization of amplifiable multicopy DNA cloning vehicles derived from the P15A cryptic miniplasmid. J Bacteriol. 1978;134(3):1141-56.

9. Bassalo MC, Garst AD, Halweg-Edwards AL, Grau WC, Domaille DW, Mutalik VK, et al. Rapid and efficient one-step metabolic pathway integration in *E. coli*. ACS Synth Biol. 2016;5(7):561-8.

10. Murphy KC, Campellone KG. Lambda Red-mediated recombinogenic engineering of enterohemorrhagic and enteropathogenic *E. coli*. BMC Mol Biol. 2003;4:11.
