# Supplementary material for: The higBA-Type Toxin-Antitoxin System in IncC Plasmids Is a Mobilizable Ciprofloxacin-Inducible System
Source: mSphere. 2021 Jun 2;6(3):e00424-21. doi: 10.1128/mSphere.00424-21 (PMC8265657; doi:10.1128/mSphere.00424-21)
Supplement: TABLE S7 [file msphere.00424-21-st007.docx]

**Table S7**

| **Bacterial strain** | **Characteristics** | **Reference** |
| --- | --- | --- |
| *E. coli* J53 | Derivative of *E. coli* K12 strain with a sodium azide resistance mutation in the *secA* gene (A112V) | (11, 12) |
| J53 + pBAD33 | J53 control strain bearing the pBAD33 vector; Gm^R^ | This study |
| J53 + pBAD33-*higB*_v1 (pJIQQ30) | J53 strain with IncC *higB* toxin gene (variant 1) in pBAD33; Gm^R^ |  |
| J53 + pBAD33-*higB*_v1_G64V | J53 strain with IncC *higB* toxin gene (variant 1) in pBAD33 with non-synonymous mutation G64V; Gm^R^ |  |
| J53 + pBAD33-*higB*_v2 (pJIQQ31) | J53 strain with IncC *higB* toxin gene (variant 2) in pBAD33; Gm^R^ |  |
| J53 + pBAD33-*higA* (pJIQQ32) | J53 strain with IncC *higA* antitoxin gene in pBAD33; Gm^R^ |  |
| J53 + pBAD33 + pBAD24 | J53 control strain with pBAD33 and pBAD24 vectors; Gm^R^ and Amp^R^ |  |
| J53 + pBAD33-*higB*_v1 (pJIQQ30) + pBAD24 | J53 strain with IncC *higB* toxin gene (variant 1) in pBAD33 and pBAD24 empty vector; Gm^R^ and Amp^R^ |  |
| J53 + pBAD33-*higB*_v1 (pJIQQ30) + pBAD24-*higA* (pJIQQ33) | J53 strain with IncC *higB* toxin gene (variant 1) in pBAD33 and *higA* antitoxin gene in pBAD24; Gm^R^ and Amp^R^ |  |
| J53-*gfpuv* | J53 derivative strain with green fluorescence protein gene *gfpuv* inserted into the SS9 chromosomal site between *aslA* and *glmZ* |  |
| J53-*gfpuv* + pACYC184 | J53-*gfpuv* control strain + pACYC184 control vector without insert; Tc^R^ and Cm^R^ |  |
| J53-*gfpuv* + pACYC184-*higBA*_v1 (pJIQQ34) | J53-*gfpuv* + pACYC184 with *higBA* TA operon promoter and operon; *higB*  (variant 1); Cm^R^ |  |
| J53-*gfpuv* + pACYC184-*higBA*_v2 (pJIQQ35) | J53-*gfpuv* + pACYC184 with *higBA* TA operon promoter and operon; *higB*  (variant 2); Cm^R^ |  |
| J53-*gfpuv* + pBAD33-*higB*_v1 (pJIQQ30) | J53-*gfpuv* with IncC *higB* toxin gene (variant 1) in pBAD33; Gm^R^ |  |
| J53-*gfpuv* + pBAD33-*higB*_v1 (G64V) | J53-*gfpuv* with IncC *higB* toxin gene (variant 1 with mutation G64V) in pBAD33; Gm^R^ |  |
| J53-*gfpuv* + pEc158ΔMDR-*tetA* | J53-*gfpuv* with MDR-deleted version of IncC clinical plasmid pEc158; Tc^R^ |  |

Abbreviations in this table for antibiotic resistance phenotypes include Gm^R^: gentamicin; Amp^R^: ampicillin; Cm^R^: chloramphenicol; Tc^R^: tetracycline; Kan^R^: kanamycin

**References**

11. Yi H, Cho YJ, Yong D, Chun J. Genome sequence of *Escherichia coli* J53, a reference strain for genetic studies. J Bacteriol. 2012;194(14):3742-3.

12. Matsumura Y, Peirano G, Pitout JDD. Complete Genome Sequence of *Escherichia coli* J53, an azide-resistant laboratory strain used for conjugation experiments. Genome Announc. 2018;6(21).
